# Summary of taxonomy changes ratified by the International Committee on Taxonomy of Viruses (ICTV) from the Bacterial Viruses Subcommittee, 2025

**DOI:** 10.1099/jgv.0.002111

**Published:** 2025-07-25

**Authors:** Dann Turner, Evelien M. Adriaenssens, Rudolf I. Amann, Pavol Bardy, Nina Bartlau, Jakub Barylski, Stanisław Błażejak, Majid Bouzari, Ariane Briegel, Yves Briers, Daniel Carrillo, Xia Chen, Dennis Claessen, Ryan Cook, Marco A. Crisci, Arnaud Dechesne, Paulina Deptula, Bas E. Dutilh, Bert Ely, Lars Fieseler, Paul C.M. Fogg, Akihito Fukudome, Mohammed Saeed Ganjoor, Iwona Gientka, Karin Holmfeldt, Panos G. Kalatzis, Kathryn M. Kauffman, Annabel Kempff, Petar Knezevic, Eugene V. Koonin, Andrew M. Kropinski, Mart Krupovic, Ipek Kurtböke, Kai Lambon, Rob Lavigne, Susan M. Lehman, H.-T. Liu, Cedric Lood, Rudi Lurz, Sari Mäntynen, Cole B. Matrishin, Mathias Middelboe, Andrew D. Millard, Cristina Moraru, Dennis S. Nielsen, Franklin L. Nobrega, Takuro Nunoura, Hanna M. Oksanen, Véronique Ongenae, Boris Parra, Célia Pas, Joseph Pogliano, Minna M. Poranen, Siravudh Potipimpanon, Amy Prichard, Hannah V. Pye, Daniela Rothschild-Rodriguez, Daniel E. Rozen, Joanne M. Santini, Yuandong Sha, Dziyana Shymialevich, Barbara Sokołowska, Abbas Soleimani-Delfan, Paulina Średnicka, Paulo Tavares, Andrea Telatin, Igor Tolstoy, Shyun-ichi Urayama, Vera van Neer, Finn K. Vogensen, Qiannan Wen, Antje Wichels, Michał Wójcicki, ICTV Taxonomy Summary Consortium, P. Alfenas-Zerbini

**Affiliations:** 1 School of Applied Sciences, Faculty of Health, Science and Society, University of the West of England, Bristol, UK; 2 Quadram Institute Bioscience, Norwich, UK; 3 Max Planck Institute for Marine Microbiology, Germany Division of Microbial Ecology, Bremen, Germany; 4 Department of Chemistry, University of York, York, UK; 5 Adam Mickiewicz University, Poznań, Poland; 6 Department of Biotechnology and Food Microbiology, Institute of Food Sciences, Warsaw University of Life Sciences (WULS-SGGW), Warsaw, Poland; 7 Department of Cell and Molecular Biology & Microbiology, Faculty of Biological Science and Technology, University of Isfahan, Isfahan, Iran; 8 Institut Pasteur, Université Paris Cité, CNRS UMR3523 Integrative Structural Cell Biology Unit, Paris, France; 9 Ghent University, Department of Biotechnology, Valentin Vaerwyckweg 1, 9000 Gent, Belgium; 10 Metagenomics Group, Utrecht University, Utrecht, Netherlands; 11 Key Laboratory of Dairy Biotechnology and Engineering, Ministry of Education, Inner Mongolia Agricultural University, 306 Zhaowuda Road, Hohhot 010018, PR China; 12 Leiden University, Institute of Biology, Leiden, Netherlands; 13 University College London, London, UK; 14 Department of Biotechnology and Biomedicine, Technical University of Denmark, Søltofts Plads, Bldg 221, 2800 Kgs. Lyngby, Denmark; 15 Department of Food Science, University of Copenhagen, Rolighedsvej 26, 1958 Frederiksberg, Denmark; 16 Institute of Biodiversity, Ecology, and Evolution, Cluster of Excellence Balance of the Microverse, Friedrich Schiller University, Jena, Germany; 17 Theoretical Biology and Bioinformatics, Science4Life, Utrecht University, 3584 CH Utrecht, Netherlands; 18 Department of Biological Sciences, University of South Carolina, Columbia, USA; 19 ZHAW School of Life Sciences and Facility Management, Centre for Food Safety and Quality Management, Einsiedlerstrasse 35, 8820 Wädenswil, Switzerland; 20 Department of Biology, University of York, York, UK; 21 Department of Biology and Department of Molecular and Cellular Biochemistry, Howard Hughes Medical Institute, Indiana University, Bloomington, IN, USA; 22 Department of Biology and Environmental Science, Faculty of Health and Life Sciences, Centre for Ecology and Evolution in Microbial Model Systems (EEMiS), Linnaeus University, Kalmar, Sweden; 23 Section for Evolutionary Hologenomics, Globe Institute, University of Copenhagen, Copenhagen, Denmark; 24 Department of Oral Biology, University at Buffalo, Buffalo, NY, USA; 25 Leiden University, Leiden, Netherlands; 26 PK Lab, Department of Biology and Ecology, Faculty of Sciences, University of Novi Sad, Novi Sad, Serbia; 27 Computational Biology Branch, Division of Intramural Research, National Library of Medicine, National Institutes of Health, Bethesda, MD, USA; 28 Department of Pathobiology, University of Guelph, Ontario, Canada; 29 Institut Pasteur, Université Paris Cité, CNRS UMR6047, Archaeal Virology Unit, Paris, France; 30 School of Science, Technology and Engineering, University of the Sunshine Coast, Sippy Downs, Australia; 31 School of Biological Sciences, University of Southampton, Southampton, UK; 32 Laboratory of Gene Technology, KU Leuven, Leuven, Belgium; 33 Center for Biologics Evaluation and Research, US Food and Drug Administration, Silver Spring, Maryland, USA; 34 Jimei University, Xiamen, PR China; 35 Department of Biology, University of Oxford, Oxford, UK; 36 Max Planck Institute for Molecular Genetics, Berlin, Germany; 37 Molecular and Integrative Biosciences Research Programme, Faculty of Biological and Environmental Sciences, University of Helsinki, Helsinki, Finland; 38 Marine Biological Section, Department of Biology, University of Copenhagen, Helsingor, Denmark; 39 Becky Mayer Centre for Phage Research, University of Leicester, University Road, Leicester, UK; 40 Carl von Ossietzky Universität Oldenburg, Oldenburg, Germany; 41 Research Center for Bioscience and Nanoscience (CeBN), Japan Agency for Marine-Earth Science and Technology(JAMSTEC), Yokosuka, Japan; 42 Departamento de Microbiología, Laboratorio de Investigación en Agentes Antibacterianos(LIAA), Facultad de Ciencias Biológicas, Universidad de Concepción, Concepción 4070409, Chile; 43 Facultad de Medicina Veterinaria y Agronomía, Instituto de Ciencias Naturales, Universidad de las Américas, Av. Jorge Alessandri 1160, Campus El Boldal, Concepción 4070409, Chile; 44 Department of Biological Sciences, University of California San Diego, La Jolla, USA; 45 Department of Microbiology, Prof. Wacław Dąbrowski Institute of Agricultural and Food Biotechnology – State Research Institute, Warsaw, Poland; 46 Université Paris-Saclay, CEA, CNRS, Institute for Integrative Biology of the Cell (I2BC), Gif-sur-Yvette, France; 47 National Center for Biotechnology Information, Bethesda, MD, USA; 48 Department of Life and Environmental Sciences, Laboratory of Fungal Interaction and Molecular Biology, University of Tsukuba, Tsukuba, Japan; 49 Alfred-Wegner Institute Helmholtz Centre for Polar and Marine Research, Biologische Anstalt Helgoland, Helgoland, Germany; 50 Department of Phage Therapy, Bacteriophage Laboratory, Hirszfeld Institute of Immunology and Experimental Therapy, Polish Academy of Sciences, Wrocław, Poland

**Keywords:** *Abidjanvirus Ab18*, *Abidjanvirus Ab19*, *Abidjanvirus PaMx11*, *Acadevirus bigMiraUFV01*, *Acadevirus premi*, *Acinetobacter virus Acj61*, *Actaeavirus*, *Actaeavirus CRP125*, *Actinidiaevirus*, *Actinidiaevirus Ep4*, *Adrianbuildvirus*, *Adrianbuildvirus ARI0923*, *Adrianbuildvirus IPP41*, *Adrianbuildvirus IPP42*, *Adrianbuildvirus IPP43*, *Adrianbuildvirus IPP44*, *Adrianbuildvirus IPP5*, *Adrianbuildvirus IPP51*, *Adrianbuildvirus SpSL1*, *Aequorvirus*, *Aequorvirus HTVC041P*, *Aeromonas virus 43*, *Ahduovirus AH2*, *Ahphunavirus A014L*, *Ahphunavirus AHPMCC7*, *Ahphunavirus LAh1*, *Ahphunavirus P2*, *Ahphunavirus ST21*, *Ahphunavirus yong1*, *Alfirinvirus*, *Alfirinvirus alfirin*, *Alisviridae*, *Alphacystovirus*, *Alphacystovirus phi8*, *Altadenavirus*, *Altadenavirus altadena*, *Altadenavirus bumble*, *Amboselivirus*, *Amboselivirus simi*, *Ameliavirus*, *Ameliavirus viph1008o*, *Anathvirus*, *Anathvirus anath*, *Andersonviridae*, *Andromedavirus leo2*, *Angmobvirus*, *Angmobvirus SCBP1*, *Anticleavirus*, *Anticleavirus jorvik*, *Apdecimavirus K12P11*, *Aristophanesvirus*, *Aristophanesvirus aristophanes*, *Armandvirus*, *Armandvirus PT2*, *Armandvirus PZLAh152*, *Armandvirus PZLAh8*, *Armandvirus T7Ah*, *Arnovirus*, *Arnovirus Wc4*, *Arnovirus arno162*, *Arnovirus arno18*, *Arthrobacter virus Liebe*, *Artimaviricota*, *Asemoviridae*, *Atoyacvirus*, *Atoyacvirus atoyac1*, *Atoyacvirus atoyac15*, *Atsuirnavirus*, *Atsuirnavirus caloris*, *Atuphduovirus atuph03*, *Autographivirales*, *Autonotataviridae*, *Autoscriptoviridae*, *Autosignataviridae*, *Autotranscriptaviridae*, *Axyvirus*, *Axyvirus 1932Axy09*, *Axyvirus 1932Axy21*, *Axyvirus 1932Axy23*, *Azeevirinae*, *Baileybluvirus callinallbarbz*, *Bajunvirus*, *Bajunvirus bajun*, *Bamvirus*, *Bamvirus bam*, *Barnstormervirus*, *Barnstormervirus barnstormer*, *Barnstormervirus caron*, *Baxterfoxvirus baxterfox*, *Baxterfoxvirus yeezy*, *Baxtervirus baxterfox*, *Baxtervirus yeezy*, *Benllochvirus*, *Benllochvirus K10PH82C1*, *Benllochvirus VLCpiA3a*, *Benllochvirus cmc355D*, *Benllochvirus cp31*, *Berlinvirus D226*, *Berlinvirus JSS1*, *Berlinvirus JSS2*, *Berlinvirus P151*, *Berlinvirus PC127*, *Berlinvirus PZJ0206*, *Berlinvirus PcCB251*, *Berlinvirus SEqdws315*, *Berlinvirus SPLA4*, *Berlinvirus SWJM03*, *Berlinvirus SalMLPST153*, *Berlinvirus V1*, *Berlinvirus Yepe2*, *Berlinvirus Yepf*, *Berlinvirus carlspitteler*, *Berlinvirus ernstbeyeler*, *Berlinvirus pEaSNUABM57*, *Berlinvirus pO103*, *Berryhillviridae*, *Bertilvirus*, *Bertilvirus bertil*, *Betacystovirus*, *Betacystovirus phi12*, *Bifilivirus philemonii*, *Bifseptvirus BIMBV45*, *Bifseptvirus SoKa*, *Boesrvirus*, *Boesrvirus BOESR1*, *Bolekvirus*, *Bolekvirus bolek*, *Bolekvirus lolek*, *Bonaevitae bonaevitae*, *Bonaevitaevirus bonaevitae*, *Bonnellvirus Kc261*, *Bonnellvirus RZ4*, *Bonnellvirus altidsur*, *Bonnellvirus glasur*, *Bonnellvirus mellemsur*, *Bonnellvirus smaasur*, *Bonnellvirus usur*, *Bordetella virus CN1*, *Bordetella virus CN2*, *Bordetella virus FP1*, *Bordetella virus MW2*, *Bronvirus bron*, *Bronvirus joedirt*, *Burkholderia virus AH2*, *Burkholderia virus BcepNazgul*, *Cafassovirus*, *Cafassovirus aleemily*, *Cafassovirus cafasso*, *Cafassovirus morgana*, *Cafassovirus obladi*, *Caliparnavirus*, *Caliparnavirus acidus*, *Campylobacter virus IBB35*, *Camvirus vanseggelen*, *Camvirus verabelle*, *Cankvirus*, *Cankvirus cv10P302A*, *Carpasinavirus FoX6*, *Casidaviridae*, *Catalonvirus*, *Catalonvirus NF1*, *Caulobacter virus Sansa*, *Cebaduodecimvirus*, *Cebaduodecimvirus phi12auna*, *Cebaduodecimvirus phi12duo*, *Ceetrepovirus C3PO*, *Ceetrepovirus darwin*, *Ceetrepovirus zion*, *Centumtrigintavirus*, *Centumtrigintavirus cv133*, *Cenunavirus*, *Cenunavirus Cen1621*, *Cepavirus*, *Cepavirus PAS7*, *Ceskevirus*, *Ceskevirus SB4*, *Chamilpavirus*, *Chamilpavirus RHEph21*, *Chemalvirus*, *Chemalvirus PseuGes254*, *Chennaivirus*, *Chennaivirus MVCVPHSA1*, *Cheoctovirus PMC*, *Cheoctovirus SG4*, *Cheoctovirus ardmore*, *Cheoctovirus boomer*, *Cheoctovirus che8*, *Cheoctovirus deadp*, *Cheoctovirus dlane*, *Cheoctovirus dorothy*, *Cheoctovirus dotproduct*, *Cheoctovirus drago*, *Cheoctovirus fruitloop*, *Cheoctovirus gumbie*, *Cheoctovirus ibhubesi*, *Cheoctovirus llij*, *Cheoctovirus mozy*, *Cheoctovirus mutaforma13*, *Cheoctovirus pacc40*, *Cheoctovirus ramsey*, *Cheoctovirus rockyhorror*, *Cheoctovirus shauna1*, *Cheoctovirus shilan*, *Cheoctovirus spartacus*, *Cheoctovirus taj*, *Cheoctovirus tweety*, *Cheoctovirus wee*, *Chimalliviridae*, *Chivirus BP12C*, *Chronisvirus*, *Chronisvirus chronis*, *Chunghsingvirus P1201*, *Colingsworthviridae*, *Conareevirus*, *Conareevirus Cd1*, *Conareevirus babayka*, *Conareevirus doublea*, *Connertonviridae*, *Cornievirus cornie*, *Corticovirus*, *Corticovirus Cr39582*, *Corticovirus PM2*, *Corycianvirus*, *Corycianvirus MfV*, *Coryciavirus*, *Coryciavirus A014S*, *Corynebacterium virus C3PO*, *Corynebacterium virus Darwin*, *Corynebacterium virus P1201*, *Corynebacterium virus Zion*, *Cotavirus*, *Cotavirus cota*, *Cronosvirus EspYZU05*, *Cronosvirus EspYZU13*, *Cronosvirus GY3*, *Cronosvirus Kc318*, *Cuernavacavirus RHEph09*, *Cuernavacavirus RHphI38*, *Cuernavacavirus RHphN37*, *Cuernavacavirus RHphTM33*, *Cullenvirus*, *Cullenvirus 6937*, *Cullenvirus K59PH2*, *Cullenvirus KYP*, *Cystovirus*, *Cystovirus phi6*, *Cystovirus phiNN*, *Dabrowskivirus*, *Dabrowskivirus KKP3916*, *Daeravirus*, *Daeravirus MA13*, *Daniellevirus*, *Daniellevirus Zyzzx*, *Daniellevirus danielle*, *Daolivirus*, *Daolivirus MJG*, *Dazunavirus*, *Dazunavirus DZ1*, *Dcimvirus*, *Dcimvirus DCM*, *Deltacystovirus*, *Deltacystovirus phi2954*, *Dewhirstvirus*, *Dewhirstvirus pging00J*, *Dewhirstvirus pging00K*, *Dewhirstvirus pging00L*, *Dewhirstvirus pging00M*, *Dexdertvirus kwekel*, *Dishuivirus*, *Dishuivirus DSLLC07*, *Divaquavirales*, *Dolichocephalovirinae*, *Dovevirinae*, *Drulisvirus BHU1*, *Drulisvirus BHU2*, *Drulisvirus BHU3*, *Drulisvirus BUCT631*, *Drulisvirus BUCT86*, *Drulisvirus Bp5*, *Drulisvirus CX1*, *Drulisvirus Dlv622*, *Drulisvirus FBKp18*, *Drulisvirus FK1979*, *Drulisvirus IME308*, *Drulisvirus IME337*, *Drulisvirus JKP2*, *Drulisvirus K15PH90*, *Drulisvirus K1PH164C1*, *Drulisvirus K24PH164C1*, *Drulisvirus K25PH129C1*, *Drulisvirus K39PH122C2*, *Drulisvirus K40PH129C1*, *Drulisvirus K51PH129C1*, *Drulisvirus K66PH128C1*, *Drulisvirus K71PH129C1*, *Drulisvirus K72PH164C2*, *Drulisvirus KA*, *Drulisvirus KMI3*, *Drulisvirus KMI6*, *Drulisvirus KPPK1081*, *Drulisvirus KPPK1082*, *Drulisvirus KPR2*, *Drulisvirus KXP*, *Drulisvirus KpV2883*, *Drulisvirus Kpn13*, *Drulisvirus LLY*, *Drulisvirus M21221*, *Drulisvirus NER40*, *Drulisvirus P1010*, *Drulisvirus P929*, *Drulisvirus PWKp1*, *Drulisvirus Pone*, *Drulisvirus QL*, *Drulisvirus SCNJ1Z*, *Drulisvirus SKP1*, *Drulisvirus SRD2021*, *Drulisvirus VAC25*, *Drulisvirus VLC1*, *Drulisvirus VLC3*, *Drulisvirus VLC4*, *Drulisvirus VLC5*, *Drulisvirus VLC6*, *Drulisvirus VLCpiA1a*, *Drulisvirus VLCpiA1b*, *Drulisvirus VLCpiA1c*, *Drulisvirus VLCpiA1d*, *Drulisvirus VLCpiA1e*, *Drulisvirus VLCpiA1f*, *Drulisvirus VLCpiA1g*, *Drulisvirus VLCpiA1h*, *Drulisvirus VLCpiA1i*, *Drulisvirus VLCpiA1j*, *Drulisvirus VLCpiA1k*, *Drulisvirus VLCpiA1l*, *Drulisvirus VLCpiA1m*, *Drulisvirus VLCpiA1n*, *Drulisvirus VLCpiA1o*, *Drulisvirus VLCpiA1q*, *Drulisvirus VLCpiA1r*, *Drulisvirus ZH5*, *Drulisvirus ZX11*, *Drulisvirus ZX6*, *Drulisvirus cp48*, *Drulisvirus dv6993*, *Drulisvirus dv6995*, *Drulisvirus fHeKpn01*, *Drulisvirus pKPM18622*, *Drulisvirus pokalde001*, *Drulisvirus tk2018*, *Drulisvirus xx20*, *Drulisvirus pKp11*, *Dunnvirinae*, *Dynamenevirus*, *Dynamenevirus CRP114*, *Dynamenevirus CRP227*, *Dynamenevirus CRP361*, *Eastwestvirus*, *Eastwestvirus eastwest*, *Ebriosvirus*, *Ebriosvirus IME15*, *Ebriosvirus ebrios*, *Edwardsroadvirus*, *Edwardsroadvirus RRH1*, *Efekovirus efeko*, *Efkovirus efeko*, *Ehrlichviridae*, *Elsinorevirus*, *Elsinorevirus NO16*, *Elunavirus PagPSK1*, *Elunavirus stepyanka*, *Emotionvirus*, *Emotionvirus emotion*, *Epseptimavirus KKP*, *Epseptimavirus KKP3831*, *Epsiloncystovirus*, *Epsiloncystovirus phiNY*, *Epsomviridae*, *Eracentumvirus Nifs112*, *Escherichia phage ESCO13*, *Escherichia virus CF2*, *Escherichia virus DE3*, *Escherichia virus ESCO5*, *Escherichia virus Schickermooser*, *Escherichia virus phAPEC8*, *Eucampyvirinae*, *Euvesivirus*, *Euvesivirus SB3*, *Excelsiorvirus*, *Excelsiorvirus pging00S*, *Felixounavirus ASO1A*, *Felixounavirus BPSELC1*, *Felixounavirus CL1*, *Felixounavirus CRP22*, *Felixounavirus CapYZU01*, *Felixounavirus D12*, *Felixounavirus DE17*, *Felixounavirus DE7*, *Felixounavirus DR094*, *Felixounavirus EC106*, *Felixounavirus ECOH1*, *Felixounavirus EF202P1*, *Felixounavirus ESCO45*, *Felixounavirus ESCO49*, *Felixounavirus ESCO50*, *Felixounavirus GSP193*, *Felixounavirus IME338*, *Felixounavirus JK55*, *Felixounavirus JLBYU28*, *Felixounavirus JLBYU32*, *Felixounavirus JN01*, *Felixounavirus KhF1*, *Felixounavirus L27*, *Felixounavirus LMP25*, *Felixounavirus MBP496116*, *Felixounavirus NBEco004*, *Felixounavirus NBEco005*, *Felixounavirus NBSal004*, *Felixounavirus NJ12*, *Felixounavirus OPTSAL01*, *Felixounavirus PHB11*, *Felixounavirus Pr103Blw*, *Felixounavirus REP5*, *Felixounavirus REP8*, *Felixounavirus RP3*, *Felixounavirus Ro111lw*, *Felixounavirus S19cd*, *Felixounavirus SEP1*, *Felixounavirus SME50*, *Felixounavirus SPJ41*, *Felixounavirus ST11*, *Felixounavirus SUTS720*, *Felixounavirus SWJM02*, *Felixounavirus Sp3Shan2021*, *Felixounavirus VSe11*, *Felixounavirus Wec171*, *Felixounavirus Z31*, *Felixounavirus ZX4221*, *Felixounavirus adrianh*, *Felixounavirus allfine*, *Felixounavirus andreotti*, *Felixounavirus barry*, *Felixounavirus bumzen*, *Felixounavirus ekra*, *Felixounavirus dune*, *Felixounavirus ev035*, *Felixounavirus ev108*, *Felixounavirus ev78*, *Felixounavirus finno*, *Felixounavirus fjerdesal*, *Felixounavirus fv1*, *Felixounavirus fv35FD*, *Felixounavirus garuso*, *Felixounavirus heid*, *Felixounavirus humlepung*, *Felixounavirus johannrwettstein*, *Felixounavirus meda*, *Felixounavirus mio*, *Felixounavirus momo*, *Felixounavirus nataliec*, *Felixounavirus pEP20*, *Felixounavirus pSJ21*, *Felixounavirus ph22*, *Felixounavirus pinkbiff*, *Felixounavirus radambza*, *Felixounavirus shy*, *Felixounavirus skuden*, *Felixounavirus tootiki*, *Felixounavirus tribble*, *Felixounavirus warpig*, *Felixviridae*, *Ferrettivirinae*, *Fibrovirus VP24*, *Firehammervirus CJLB12*, *Firehammervirus CJLB14*, *Firehammervirus CJLB15*, *Firehammervirus F379*, *Fletchervirus CJLB10*, *Fletchervirus CJLB7*, *Fletchervirus F207*, *Fletchervirus F336*, *Fletchervirus F341*, *Fletchervirus F372*, *Fletchervirus PC5*, *Fletchervirus QDYZ*, *Fobrovirus VP24*, *Foetvirus P1723*, *Foturvirus R8W*, *Frickvirinae*, *Friunavirus 3043K38*, *Friunavirus AB3*, *Friunavirus ABSZ6*, *Friunavirus ABWU2101*, *Friunavirus AGC01*, *Friunavirus AIIMSAbE5RC*, *Friunavirus APK09*, *Friunavirus APK116*, *Friunavirus APK127v*, *Friunavirus APK128*, *Friunavirus APK14*, *Friunavirus APK15*, *Friunavirus APK16*, *Friunavirus APK2*, *Friunavirus APK20*, *Friunavirus APK26*, *Friunavirus APK32*, *Friunavirus APK37*, *Friunavirus APK371*, *Friunavirus APK48*, *Friunavirus APK483*, *Friunavirus APK77*, *Friunavirus APK81*, *Friunavirus APK86*, *Friunavirus APK87*, *Friunavirus APK89*, *Friunavirus AbP7*, *Friunavirus AbTP31*, *Friunavirus AbpL*, *Friunavirus Acba6*, *Friunavirus ChT04*, *Friunavirus F70K44*, *Friunavirus Hep4*, *Friunavirus IME546*, *Friunavirus MRABP9*, *Friunavirus P1489*, *Friunavirus PMK34*, *Friunavirus Paty*, *Friunavirus Pipo*, *Friunavirus SWHAb1*, *Friunavirus SWHAb3*, *Friunavirus WU2001*, *Friunavirus YZ2*, *Friunavirus ZHSHW*, *Friunavirus fBenAci001*, *Friunavirus fBenAci002*, *Friunavirus fBenAci003*, *Friunavirus pB3074*, *Fujianvirus*, *Fujianvirus V141*, *Furtirnaviricetes*, *Fussvirus eyrgjafa*, *Fuzzbustervirus*, *Fuzzbustervirus fuzzbuster*, *Gajwadongvirus MR4*, *Gammacystovirus*, *Gammacystovirus phi13*, *Gammacystovirus phiYY*, *Gansuvirus*, *Gansuvirus F4M1D*, *Gansuvirus F5M1D*, *Gansuvirus FBKp16*, *Gansuvirus K13PH07C1L*, *Gansuvirus K22PH164C1*, *Gansuvirus K61PH164C1*, *Gansuvirus KPN7*, *Gansuvirus Kp7*, *Gansuvirus PmP19*, *Gansuvirus VLCpiA2a*, *Gansuvirus VLCpiA2b*, *Gansuvirus ZK1*, *Gansuvirus pKVBS37531*, *Gardenstatevirus*, *Gardenstatevirus gardenstate*, *Gardenstatevirus iamgroot*, *Gettysburgvirus*, *Gettysburgvirus gv019DV002*, *Gettysburgvirus gv056SW001B*, *Gettysburgvirus gv268TH004*, *Ghunavirus17A*, *Ghunavirus AH05*, *Ghunavirus CHF1*, *Ghunavirus CHF7*, *Ghunavirus PCW2*, *Ghunavirus Psa17*, *Ghunavirus PstGIL1*, *Ghunavirus athelas*, *Ghunavirus gv17A*, *Gladiolivirus*, *Gladiolivirus maja*, *Gordonia virus Lennon*, *Gordonia virus Yvonnetastic*, *Grandevirales*, *Grimontviridae*, *Guangxivirus*, *Guangxivirus PSTH2*, *Gujervirinae*, *Gundecimvirus*, *Gundecimvirus MG11*, *Gyeongsanvirus PPSG11*, *Haasevirus*, *Haasevirus pging00R*, *Haasevirus pging00T*, *Haasevirus pging00U*, *Haasevirus pging00V*, *Haasevirus pging00W*, *Hakuzoviridae*, *Hapakavirus*, *Hapakavirus Nufs112*, *Hatfieldvirus*, *Hatfieldvirus porci*, *Helsettvirus YpEc11*, *Helsettvirus fPS53*, *Hennigervirus*, *Hennigervirus MR1*, *Hennigervirus MR2*, *Hennigervirus PPPL1*, *Hennigervirus henninger*, *Hennigervirus shl2*, *Higashivirus BHDTSo9*, *Hilgardvirus*, *Hilgardvirus vroomvroom*, *Hinxtonvirus*, *Hinxtonvirus ARI0004*, *Hinxtonvirus ARI0031*, *Hinxtonvirus ARI02851*, *Hinxtonvirus ARI0462*, *Hinxtonvirus ARI04681*, *Hinxtonvirus ARI0468b3*, *Hinxtonvirus ARI0831b*, *Hinxtonvirus DCC1738*, *Hinxtonvirus IC1*, *Hinxtonvirus IPP34*, *Hinxtonvirus IPP46*, *Hinxtonvirus IPP64*, *Hinxtonvirus IPP69*, *Hinxtonvirus K13*, *Hinxtonvirus V22*, *Hinxtonvirus hv2167*, *Hinxtonvirus hv34117*, *Hinxtonvirus hv8140*, *Hodgkinviridae*, *Hongshanvirus*, *Hongshanvirus BMB50*, *Honkvirus*, *Honkvirus honk*, *Honmavirus*, *Honmavirus pging00B*, *Honmavirus pging00C*, *Honmavirus pging00D*, *Honmavirus pging00E*, *Honmavirus pging00F*, *Honmavirus pging00G*, *Honmavirus pging00H*, *Honmavirus pging00I*, *Irusalimvirus*, *Irusalimvirus BCSR52*, *Jawnskivirus*, *Jawnskivirus beans*, *Jawnskivirus brent*, *Jawnskivirus jawnski*, *Jawnskivirus king2*, *Jawnskivirus piccoletto*, *Jeanschmidtviridae*, *Jelgvirus*, *Jelgvirus JELGKS1*, *Jeruvirus*, *Jeruvirus PSTNGR1*, *Jiaweivirus*, *Jiaweivirus jiawei*, *Jimeivirus*, *Jimeivirus LHP*, *Jinkiesvirus*, *Jinkiesvirus jinkies*, *Kakivirus PSTRCR114*, *Kaohsiungvirus AS51*, *Kaohsiungvirus MGD2*, *Kaohsiungvirus R15Z*, *Kaohsiungvirus VPHS15*, *Kaohsiungvirus VaZX1*, *Karimacvirus karimac*, *Karimacvirus lukecage*, *Karimacvirus starplatinum*, *Karimacvirus wofford*, *Karimacvirus yaboi*, *Kayfunavirus101118UKE1*, *Kayfunavirus 216Ecol046PP*, *Kayfunavirus22664UKE32*, *Kayfunavirus 6925*, *Kayfunavirus B1*, *Kayfunavirus CLBP1*, *Kayfunavirus CY1*, *Kayfunavirus EP1*, *Kayfunavirus EPr2*, *Kayfunavirus EV1361*, *Kayfunavirus EcoPRo103C3lw*, *Kayfunavirus EcpYZU01*, *Kayfunavirus HC12*, *Kayfunavirus HC13*, *Kayfunavirus IME177*, *Kayfunavirus IME278*, *Kayfunavirus IMEP24*, *Kayfunavirus KKP3263*, *Kayfunavirus Kc166A*, *Kayfunavirus LET1*, *Kayfunavirus LS2*, *Kayfunavirus LS3*, *Kayfunavirus Mt1B1P3*, *Kayfunavirus NS1*, *Kayfunavirus P762*, *Kayfunavirus PH1061*, *Kayfunavirus PRFSP1*, *Kayfunavirus R1*, *Kayfunavirus SFP20*, *Kayfunavirus SFP21A*, *Kayfunavirus SFP21B*, *Kayfunavirus SP7*, *Kayfunavirus SR04*, *Kayfunavirus ST10*, *Kayfunavirus ST15*, *Kayfunavirus ST16*, *Kayfunavirus ST17*, *Kayfunavirus ST20*, *Kayfunavirus ST21*, *Kayfunavirus ST57*, *Kayfunavirus TM1*, *Kayfunavirus U8*, *Kayfunavirus ZH4*, *Kayfunavirus emlis*, *Kayfunavirus midid*, *Kayfunavirus milel*, *Kayfunavirus p02*, *Kayfunavirus pO91*, *Kayfunavirus peacock*, *Kayfunavirus penshu1*, *Kayfunavirus pisces*, *Kayfunavirus yong1*, *Kayfunavirus zappy*, *Kikimoravirus*, *Kikimoravirus gurke*, *Kikimoravirus kikimora*, *Klebsiella virus ZCKP1*, *Kolesnikvirus SE5*, *Kononvirus*, *Kononvirus KKP3711*, *Kotilavirus CX5*, *Kotilavirus MA2*, *Koutsourovirus EhYP*, *Koutsourovirus KKP3828*, *Koutsourovirus Pec*, *Kozievirus MO526*, *Kronosvirus*, *Kronosvirus elgin*, *Kronosvirus pelion*, *Kronosvirus pomeria*, *Kruegerviridae*, *Kuravirus LAMP*, *Kuravirus SDYTW1F1223*, *Kuravirus SR02*, *Kuravirus XT18*, *Kuravirus YF01*, *Kuravirus myPSH1131*, *Kuravirus myPSH2311*, *Kuravirus pECN12032Af1*, *Kuwvirus*, *Kuwvirus ParKuw1*, *Lacfervirus*, *Lacfervirus LFP01*, *Lakviridae*, *Lambdavirus DE3*, *Lambovirus birthdayboy*, *Lambovirus erutan*, *Lambovirus fulcrum*, *Lambovirus genamy16*, *Lambovirus jalebi*, *Lambovirus novasharks*, *Lambovirus otterstedtS21*, *Lambovirus parvustarda*, *Lambovirus patos*, *Lambovirus wojtek*, *Lambovirus zany*, *Lasallevirus Acj61*, *Lavrentieva E21*, *Lavrentieva PM87*, *Lavrentieva pPM01*, *Lavrentievavirus E21*, *Lavrentievavirus PM87*, *Lavrentievavirus pPM01*, *Leonardvirus phauci*, *Liebevirus liebe*, *Liebevirus maguco*, *Liefievirus halo*, *Liefievirus liefie*, *Lilmacvirus*, *Lilmacvirus bolt007*, *Lilmacvirus klevey*, *Lilmacvirus lilmac1015*, *Lilmacvirus prairie*, *Lindbergviridae*, *Linggongvirus*, *Linggongvirus VH5*, *Lomovskayavirus shawty*, *Loughboroughvirus ZCSE2*, *Lucadorvirus gail*, *Lucadorvirus jeeves*, *Lucadorvirus luchador*, *Luchadorvirus gail*, *Luchadorvirus jeeves*, *Luchadorvirus luchador*, *Ludisviridae*, *Ludisvirus*, *Ludisvirus pging00A*, *Lullwatervirus quill52*, *Lundtoftevirus*, *Lundtoftevirus Lu221*, *Luzcentumvirus*, *Luzcentumvirus LUZ100*, *Mabodamacavirus*, *Mabodamacavirus mabodamaca*, *Maculvirus AC2*, *Maculvirus BUCT233*, *Maculvirus DE10*, *Maculvirus DE17*, *Maculvirus DE18*, *Maculvirus F23s2*, *Maculvirus FE11*, *Maculvirus GHSM17*, *Maculvirus H256D1*, *Maculvirus HA1*, *Maculvirus HA5*, *Maculvirus MGD1*, *Maculvirus OWB*, *Maculvirus SrVc9*, *Maculvirus VP9*, *Maevirinae*, *Maklayavirus*, *Maklayavirus Q19*, *Malkevirus*, *Malkevirus ARI02853*, *Malkevirus IPP45*, *Malkevirus IPP67*, *Malkevirus mv11865*, *Malkevirus mv23782*, *Mallvirus*, *Mallvirus ParMal1*, *Maltophvirus*, *Maltophvirus BUCT609*, *Manhattanvirus vresidence*, *Manhattanvirus wildwest*, *Marchewkavirus*, *Marchewkavirus domovoi*, *Marchewkavirus kabachok*, *Marchewkavirus marchewka*, *Margaeryvirus*, *Margaeryvirus margaery*, *Margaeryvirus terij*, *Mazoviaviridae*, *Mboduovirus*, *Mboduovirus mbo2*, *Mcshanvirinae*, *Medawarvirus*, *Medawarvirus ARI01312*, *Medawarvirus IPP11*, *Medawarvirus IPP12*, *Medawarvirus IPP17*, *Medawarvirus IPP18*, *Medawarvirus IPP19*, *Medawarvirus IPP20*, *Medawarvirus IPP21*, *Medawarvirus IPP22*, *Medawarvirus IPP28*, *Medawarvirus IPP29*, *Medawarvirus IPP30*, *Medawarvirus IPP57*, *Medawarvirus IPP63*, *Meganvirus nichole72*, *Melbournevirus*, *Melbournevirus REQ2*, *Mengvirus*, *Mengvirus NMeng1*, *Merivirus*, *Merivirus Cr39582*, *Merivirus PM2*, *Mestraviridae*, *Micantvirus*, *Micantvirus loshitsa2*, *Micantvirus micant*, *Minipunavirus lilpapawes*, *Mojovirus*, *Mojovirus MR5*, *Mooglevirus CHB7*, *Mooglevirus EP1*, *Mooglevirus HP1*, *Mooglevirus Henu11*, *Mooglevirus KMM2*, *Mooglevirus KMM4*, *Mooglevirus M7196WT1*, *Mooglevirus PC7913*, *Mooglevirus SFPB*, *Mooglevirus mistaenkt*, *Mooglevirus silverhawkium*, *Mooglevirus susp1*, *Mooglevirus susp2*, *Morelosvirus*, *Morelosvirus RHphI20*, *Mosigvirus*, *Mosigvirus efftwo*, *Mosigvirus jaykay*, *Mtkvariviridae*, *Murciavirus CB5A*, *Mycobacterium virus Ardmore*, *Mycobacterium virus Boomer*, *Mycobacterium virus Bron*, *Mycobacterium virus Che8*, *Mycobacterium virus Cornie*, *Mycobacterium virus DeadP*, *Mycobacterium virus Dlane*, *Mycobacterium virus Dorothy*, *Mycobacterium virus DotProduct*, *Mycobacterium virus Drago*, *Mycobacterium virus Fruitloop*, *Mycobacterium virus GUmbie*, *Mycobacterium virus Halo*, *Mycobacterium virus Ibhubesi*, *Mycobacterium virus JoeDirt*, *Mycobacterium virus Liefie*, *Mycobacterium virus Llij*, *Mycobacterium virus Mozy*, *Mycobacterium virus Mutaforma13*, *Mycobacterium virus PMC*, *Mycobacterium virus Pacc40*, *Mycobacterium virus Ramsey*, *Mycobacterium virus Renaud18*, *Mycobacterium virus RockyHorror*, *Mycobacterium virus SG4*, *Mycobacterium virus Shauna1*, *Mycobacterium virus Shilan*, *Mycobacterium virus Spartacus*, *Mycobacterium virus Squirty*, *Mycobacterium virus TChen*, *Mycobacterium virus TM4*, *Mycobacterium virus Taj*, *Mycobacterium virus ThetaBob*, *Mycobacterium virus Tortellini*, *Mycobacterium virus Tweety*, *Mycobacterium virus Wee*, *Mycobacterium virus Wildcat*, *Myosmarvirus SMP*, *Myranavirus phabba*, *Myrnavirus phabba*, *Nairobivirus*, *Nairobivirus nv36*, *Nakavirus*, *Nakavirus sapi*, *Natansvirus*, *Natansvirus SnaPR1*, *Nazgulvirus bcepnazgul*, *Nerivirus*, *Nerivirus SSRP01*, *Nerthusvirus*, *Nerthusvirus BUCT553*, *Nerthusvirus achelous*, *Nerthusvirus alpheus*, *Nerthusvirus nerthus*, *Ningirsuvirus DchS19*, *Ningirsuvirus W2B*, *Ningirsuvirus nahilimali*, *Ningirsuvirus pEpSNUABM12*, *Ningirsuvirus sibilus*, *Nixviridae*, *Nixvirus*, *Nixvirus pging00X*, *Njordvirus*, *Njordvirus njord*, *Norfolkplacevirus*, *Norfolkplacevirus ARI0746*, *Norfolkplacevirus IPP15*, *Novosibovirus 309*, *Novosibovirus RS1pmA*, *Novosibovirus RS8pmA*, *Obscuriviridae*, *Oceanidvirus*, *Oceanidvirus CRP113*, *Oceanidvirus CRP171*, *Oliviavirus*, *Oliviavirus viph1020o*, *Omahavirus*, *Omahavirus UNOG1W1*, *Omtjevirus*, *Omtjevirus Omtje*, *Orthocystovirus*, *Orthocystovirus phi6*, *Orthocystovirus phiNN*, *Paadamvirus TRX321*, *Pakuvirus*, *Pakuvirus paku*, *Palovirus*, *Palovirus palo*, *Pantevenvirales*, *Parnassusviridae*, *Pasovirus*, *Pasovirus paso*, *Pastovirus*, *Pastovirus pasto*, *Paternavirus*, *Paternavirus doca7*, *Pazvirus*, *Pazvirus 31*, *Pazvirus paz*, *Paulavirus viph1008o*, *Paulavirus viph1020o*, *Pbunavirus DL68*, *Pbunavirus FBPa14*, *Pbunavirus FBPa35*, *Pbunavirus JG024*, *Pbunavirus NH4*, *Pbunavirus PSA09*, *Pbunavirus PSA25*, *Pbunavirus SG1*, *Pbunavirus TH15*, *Pbunavirus ph0031*, *Pbunavirus pv109*, *Pbunavirus victoria*, *Pbunavirus wadjak13*, *Pektosvirus PP81*, *Percivalvirus*, *Percivalvirus floof*, *Percivalvirus percival*, *Percyvirus BL198*, *Percyvirus ERS*, *Percyvirus KSC*, *Pfluvirus*, *Pfluvirus PFP1*, *Pfluvirus pv22PfluR64PP*, *Phadecavirus*, *Phadecavirus PH10*, *Phadecavirus olisA1*, *Phadecavirus pv23TH*, *Phapecoctavirus ESCO13*, *Phapecoctavirus ESCO5*, *Phapecoctavirus ZCKP1*, *Phapecoctavirus phAPEC8*, *Phapecoctavirus schickermooser*, *Phikmvvirus551W*, *Phikmvvirus AIIMSPaA1*, *Phikmvvirus FBPa3*, *Phikmvvirus HX1*, *Phikmvvirus JB10*, *Phikmvvirus MYY9*, *Phikmvvirus NFS*, *Phikmvvirus P1G*, *Phikmvvirus PA69*, *Phikmvvirus PE3*, *Phikmvvirus PJNP013*, *Phikmvvirus PT2*, *Phikmvvirus S1*, *Phikmvvirus SB*, *Phikmvvirus SEMA*, *Phikmvvirus phipa2*, *Phikmvvirus pv401*, *Phrappuccinovirus phrappuccino*, *Phreappuccinovirus Phrappuccino*, *Phutvirus PSA6*, *Pifdecavirus BIMBV46*, *Pijolavirus Pf17397FPD1*, *Pijolavirus UFJFPfSW6*, *Plutovirus*, *Plutovirus pluto*, *Pollyceevirus Eisa9*, *Polsinellivirinae*, *Polymedevirus*, *Ponderosavirus*, *Polymedevirus YY*, *Ponderosavirus SB2*, *Ponderosavirus TS10*, *Ponderosavirus pepon*, *Ponderosavirus ponderosa*, *Ponderosavirus ptah*, *Pradovirus F5*, *Pradovirus IVIADoCa2*, *Pradovirus IVIADoCa4*, *Pradovirus IVIADoCa9*, *Pradovirus MUD8T1*, *Pradovirus NED111*, *Pradovirus P4*, *Pradovirus SB5*, *Pradovirus pXoo2106*, *Pradovirus pagan*, *Pradovirus titanX*, *Proddevirus*, *Proddevirus proddE*, *Prospektnaukivirus*, *Prospektnaukivirus sam112*, *Przondovirus 066022*, *Przondovirus 066046*, *Przondovirus 175029*, *Przondovirus 2146HW*, *Przondovirus Adeo*, *Przondovirus AmPhEK52*, *Przondovirus BUCT3589*, *Przondovirus EKp2*, *Przondovirus FZ12*, *Przondovirus GWPA139*, *Przondovirus GWPB35*, *Przondovirus H5*, *Przondovirus HZJ31*, *Przondovirus IME264*, *Przondovirus IME335*, *Przondovirus IME531*, *Przondovirus K16PH164C3*, *Przondovirus JB48*, *Przondovirus K11PH164C1*, *Przondovirus K2044302*, *Przondovirus K2044HW*, *Przondovirus K26PH128C1*, *Przondovirus K27PH129C1*, *Przondovirus K35PH164C3*, *Przondovirus K37PH164C1*, *Przondovirus K42PH8*, *Przondovirus K48PH164C1*, *Przondovirus K56PH164C1*, *Przondovirus K58PH129C2*, *Przondovirus K74PH129C2*, *Przondovirus K80PH1317a*, *Przondovirus K8PH128*, *Przondovirus KKP3708*, *Przondovirus KMI1*, *Przondovirus KMI2*, *Przondovirus KMI4*, *Przondovirus KPN3*, *Przondovirus Kp11*, *Przondovirus Kp9*, *Przondovirus KpV766*, *Przondovirus KpV92*, *Przondovirus Kpn17*, *Przondovirus KpnP1*, *Przondovirus KundULIP47*, *Przondovirus KundULIP54*, *Przondovirus MUC100*, *Przondovirus NK20*, *Przondovirus NLZS1*, *Przondovirus NLZS2*, *Przondovirus P509*, *Przondovirus P510*, *Przondovirus P55*, *Przondovirus P560*, *Przondovirus P671*, *Przondovirus P791*, *Przondovirus SHKP152226*, *Przondovirus TUN1*, *Przondovirus VAC71*, *Przondovirus VLCpiA3b*, *Przondovirus VLCpiA3c*, *Przondovirus VLCpiA3d*, *Przondovirus W14TH13021*, *Przondovirus ZK2*, *Przondovirus ZX10*, *Przondovirus ZX8*, *Przondovirus cmc356*, *Przondovirus cornelius*, *Przondovirus cp10*, *Przondovirus cp11*, *Przondovirus cp12*, *Przondovirus cp26*, *Przondovirus cp29*, *Przondovirus cp30*, *Przondovirus cp33*, *Przondovirus cp43*, *Przondovirus cp6*, *Przondovirus cp7*, *Przondovirus cp8*, *Przondovirus pokalde002*, *Przondovirus pv066013*, *Przondovirus pv066023*, *Przondovirus pv066024*, *Przondovirus pv066037*, *Przondovirus pv066042*, *Przondovirus pv066053*, *Przondovirus pv066056*, *Przondovirus pv117*, *Przondovirus pv150004*, *Przondovirus pv175003*, *Przondovirus pv175005*, *Przondovirus pv175006*, *Przondovirus pv175007*, *Przondovirus pv175017*, *Przondovirus pv175019*, *Przondovirus pv175022*, *Przondovirus pv175024*, *Przondovirus pv175026*, *Przondovirus pv175032*, *Pseudomonas virus Ab18*, *Pseudomonas virus Ab19*, *Pseudomonas virus LKO4*, *Pseudomonas virus DL68*, *Pseudomonas virus JG024*, *Pseudomonas virus M6*, *Pseudomonas virus MP1412*, *Pseudomonas virus NH4*, *Pseudomonas virus PAE1*, *Pseudomonas virus PaMx11*, *Pseudomonas virus Yua*, *Punavirus pv43*, *Quadringentisvirinae*, *Quhwahvirus littlefortune*, *Quhwahvirus pulchra*, *Quingentivirinae*, *Rambovirus*, *Rambovirus rambo*, *Reminisvirus*, *Reminisvirus 6939*, *Reminisvirus KL01*, *Reminisvirus reminis*, *Reqipinevirus*, *Reqipinevirus reqipine5*, *Reynauldvirus*, *Reynauldvirus reynauld*, *Rindgevirus*, *Rindgevirus tarrare*, *Risoevirus*, *Risoevirus cronus*, *Rivavirus*, *Rivavirus SPP1*, *Rivavirus rv000TH010*, *Rivavirus rv049ML001*, *Rodentiumvirus*, *Rodentiumvirus CrRp3*, *Rodentiumvirus LL11*, *Rodentiumvirus NTNC80A*, *Rodentiumvirus P101117UKE2*, *Rodentiumvirus PP433*, *Rodentiumvirus R4596*, *Roscoffvirus*, *Roscoffvirus rv15E36*, *Rosemountvirus ZCSE2*, *Roskildevirus cronus*, *Roskildevirus*, *Rumoivirus*, *Rumoivirus VruC*, *Salmonella virus BP12C*, *Sanovirus sano*, *Sansavirus sansa*, *Sarkviridae*, *Sarmavirus*, *Sarmavirus sarma103*, *Savitribaivirus*, *Savitribaivirus PS*, *Schenleyvirinae*, *Schifferlevirus*, *Schifferlevirus pging00N*, *Schifferlevirus pging00O*, *Schifferlevirus pging00P*, *Schifferlevirus pging00Q*, *Sebastisaurusvirus*, *Sebastisaurusvirus heather*, *Sebastisaurusvirus remusloopin*, *Sebastisaurusvirus sebastisaurus*, *Sechaudvirinae*, *Sepahanvirus*, *Sepahanvirus GN1*, *Sepahanvirus yerA41*, *Serkorvirus 10RS306A*, *Serkorvirus RpY2*, *Serkorvirus p2106*, *Sescentorumvirinae*, *Shadyvirus*, *Shadyvirus shady*, *Shaekyvirus*, *Shaekyvirus shaeky*, *Shangxiadianvirus*, *Shangxiadianvirus CRP118*, *Shangxiadianvirus CRP403*, *Sharonstreetvirus xiamensis*, *Sicariusvirus*, *Sicariusvirus sicarius2*, *Sicariusvirus wyborn*, *Smasvirus*, *Smasvirus BUCT598*, *Smasvirus BUCT700*, *Smasvirus P15*, *Smasvirus SB1*, *Smasvirus c9N*, *Snaubvirus*, *Snaubvirus pEpSNUABM09*, *Solymavirus*, *Solymavirus PSTCR2*, *Solymavirus PSTRCR120*, *Songlingvirus*, *Songlingvirus SI01*, *Soropartiviridae*, *Spinunavirus*, *Spinunavirus ARI01311*, *Spinunavirus ARI0399*, *Spinunavirus ARI04601*, *Spinunavirus ARI04602*, *Spinunavirus ARI04682*, *Spinunavirus IPP10*, *Spinunavirus IPP31*, *Spinunavirus IPP32*, *Spinunavirus IPP36*, *Spinunavirus IPP37*, *Spinunavirus IPP50*, *Spinunavirus IPP60*, *Spinunavirus IPP61*, *Spinunavirus IPP8*, *Spinunavirus IPP9*, *Spinunavirus SF39*, *Spinunavirus Spn1*, *Spinunavirus sv040922*, *Spiovirus*, *Spiovirus sbp1*, *Splendidredvirus*, *Splendidredvirus ray17*, *Splendidredvirus splendidred*, *Squirtyvirus squirty*, *Stackebrandtviridae*, *Stentvirinae*, *Streptomyces virus Karimac*, *Streptomyces virus LukeCage*, *Streptomyces virus StarPlatinum*, *Streptomyces virus Wollford*, *Streptomyces virus Yaboi*, *Sumtervirus*, *Sumtervirus S2B*, *Suseptimavirus PAS59*, *Suseptimavirus sv4E8*, *Suspvirus*, *Suttonboningtonvirus*, *Suttonboningtonvirus sv1ICo2020*, *Swepdovirus*, *Swepdovirus SWEP2*, *Sycamorevirus*, *Sycamorevirus sycamore*, *Tartuvirus*, *Tartuvirus amme3*, *Tartuvirus kopa4*, *Tartuvirus nopa*, *Tartuvirus roomu2*, *Teetrevirus CT02*, *Teetrevirus EF2*, *Teetrevirus Emp27*, *Teetrevirus F1M1C*, *Teetrevirus F2M1C*, *Teetrevirus F5M1C*, *Teetrevirus HMGUsm2*, *Teetrevirus IME305*, *Teetrevirus K19PH14C4P1*, *Teetrevirus KP13MC52*, *Teetrevirus Kp31*, *Teetrevirus Kpn11mx*, *Teetrevirus KpnPVAC1*, *Teetrevirus NLZS3*, *Teetrevirus Seera*, *Teetrevirus SALSA*, *Teetrevirus T7M*, *Teetrevirus bumbleweed*, *Teetrevirus glastosback*, *Teetrevirus keithsmous*, *Teetrevirus keithstache*, *Teetrevirus megaducksbill*, *Teetrevirus mtp14*, *Teetrevirus mtp15*, *Teetrevirus mtp4*, *Teetrevirus mtp8*, *Teetrevirus patroon*, *Teetrevirus sekstaphage1*, *Teetrevirus tv066044*, *Teetrevirus tv31*, *Teetrevirus tv6996*, *Teetrevirus tv6998*, *Teetrevirus vB8388*, *Tepoztlanvirus*, *Tepoztlanvirus RHphTM34*, *Tepoztlanvirus RHphY120*, *Tequatrovirus*, *Tequatrovirus CF2*, *Teseptimavirus A1122*, *Teseptimavirus C2*, *Teseptimavirus EFA2*, *Teseptimavirus JB01*, *Teseptimavirus PHB19*, *Teseptimavirus PVN09*, *Teseptimavirus SYGE1*, *Teseptimavirus UFV01*, *Teseptimavirus YpPY*, *Teseptimavirus YpYeO9*, *Teseptimavirus YpsPG*, *Teseptimavirus jacobburckhardt*, *Teseptimavirus jeantinguely*, *Teseptimavirus pO111*, *Teseptimavirus pila*, *Teseptimavirus tv04922B*, *Teseptimavirus yanou*, *Tevenvirinae*, *Thetabobvirus renaud18*, *Thetabobvirus tchen*, *Thetabobvirus thetabob*, *Thoosavirus*, *Thoosavirus HTVC025P*, *Timquatrovirus TM4*, *Tiranvirus*, *Tiranvirus STIP28*, *Tolavirus*, *Tolavirus tola*, *Torinorumvirus*, *Torinovirus*, *Torinorumvirus B11*, *Torinovirus K7A1*, *Tortellinivirus tortellini*, *Trautnerviridae*, *Trelivelvirus*, *Trelivelvirus VAC51*, *Triteiavirus*, *Triteiavirus CRP804*, *Troedvirus stalingrad*, *Trogglehumpervirus*, *Trogglehumpervirus trogglehumper*, *Tuaanevirus ime13*, *Tulanevirus ime13*, *Tunggulvirus GM223*, *Uavernvirus*, *Uavernvirus uavern*, *Uetakevirus PAS61*, *Ulipvirus*, *Ulipvirus IME184*, *Ulipvirus K1ULIP33*, *Ulipvirus K44PH129C1*, *Ulipvirus cp4*, *Ulipvirus pKPM18621*, *Unosvirus*, *Unosvirus PCS4*, *Unosvirus UNOSLW1*, *Unyawovirus PC2B6*, *Vandenendeviridae*, *Vashvirus euratis*, *Vectrevirus EC120*, *Vectrevirus EEc2*, *Vectrevirus KAW1A4500*, *Vectrevirus Mt1B1P10*, *Vectrevirus P101101UKE1*, *Vectrevirus PUTI89UKE2*, *Vectrevirus ULINTec4*, *Vectrevirus ULINTec6*, *Vectrevirus ULINTec7*, *Vectrevirus cee*, *Vectrevirus vv6948*, *Ventosusvirus*, *Ventosusvirus ventosus*, *Vetruanivirus*, *Vetruanivirus porcinsecundi*, *Vetruanivirus dhakaense*, *Vetruanivirus porcinprimi*, *Vetruanivirus primi*, *Vetruanivirus secundi*, *Vinavirales*, *Vistulavirus*, *Vistulavirus BB8*, *Vividuovirus lennon*, *Vividuovirus sitar*, *Vojvodinavirus CN1*, *Waldovirus*, *Vojvodinavirus CN2*, *Vojvodinavirus FP1*, *Vojvodinavirus MW2*, *Waldovirus plaquesplease*, *Waldovirus waldo5*, *Warsawvirus 3MF5*, *Warsawvirus wv3MF5*, *Weillhallvirus*, *Weillhallvirus wv16Q*, *Wendovervirus*, *Wendovervirus sonii*, *Wifcevirus AV128*, *Wifcevirus EC150*, *Wifcevirus ECO71P1*, *Wifcevirus Ro157lw*, *Wifcevirus SP13*, *Wifcevirus mansfield*, *Wildcatvirus wildcat*, *Wizardvirus halo3*, *Wodongavirus*, *Wodongavirus REQ3*, *Wuhanvirus PS07*, *Xeniaduovirus*, *Xeniaduovirus xenia2*, *Xylella virus Sano*, *Yangvirus ascela*, *Yangvirus berrie*, *Yangvirus cassia*, *Yangvirus janeemi*, *Yangvirus nitro*, *Yangvirus tfortroy*, *Yangvirus tuck*, *Yautepecvirus*, *Yautepecvirus RHphX39*, *Yinyavirus*, *Yinyavirus AerP220*, *Yuanmingyuanvirus*, *Yuanmingyuanvirus NJ2*, *Yuavirus LKO4*, *Yuavirus M6*, *Yuavirus MP1412*, *Yuavirus PAE1*, *Yuavirus yua*, *Yunamivirus*, *Yunamivirus Y1MI*, *Yvonnevirus yvonnetastic*, *Zetacystovirus*, *Zetacystovirus CAP*, *Zindervirus GRNsp51*, *Zindervirus kenyaK30*, *Zitchvirus apunk*, *Zitchvirus sampson*, *Zitchvirus tardus*, *Zitchvirus viaconlectus*, *Zoomievirus*, *Zoomievirus zoomie*

## Abstract

This article summarises the activities of the International Committee on Taxonomy of Viruses Bacterial Viruses Subcommittee, detailing developments in the classification of bacterial viruses. We provide here an overview of all new, abolished, moved and renamed taxa proposed in 2024, approved by the Executive Committee, and ratified by membership vote in 2025. Through the collective efforts of 74 international contributors of taxonomy proposals in this round, 43 ratified proposals have led to the creation of one new phylum, one class, four orders, 33 families, 14 subfamilies, 194 genera and 995 species. These proposals mark significant progress in refining the taxonomy of bacterial viruses. Key updates include the creation of new orders and families that include existing taxa to better reflect genomic and evolutionary relationships. As sequencing and bioinformatics approaches continue to advance, further expansion and refinements in viral taxonomy can be anticipated in the coming years.

## Introduction

The taxonomy of bacterial viruses has undergone significant changes in recent years, notably the abolishment of the paraphyletic families *Myoviridae*, *Siphoviridae* and *Podoviridae*, along with the order *Caudovirales* [1,2]. This reorganization left a number of orphan subfamilies and genera within the class *Caudoviricetes* without a designated family or order. The adoption of genome-based classification, which focuses on reconstructing virus evolutionary histories through nucleotide and amino-acid sequence similarity, phylogenetic analysis of core genes and increasingly, of protein folds and motifs for the demarcation of higher ranks, has enabled the identification of many new monophyletic taxa [3]. Additionally, a number of tools are now available to aid the classification of bacterial viruses across different taxonomic ranks (e.g. [4,7]).

Here, we present summaries of all ratified taxonomic proposals submitted to the Bacterial Viruses Subcommittee of the International Committee on Taxonomy of Viruses (ICTV) in 2024. These proposals were assessed and approved at the ICTV Executive Committee meeting (EC56, July 2024) and ratified by membership vote in 2025. Full versions of the proposals summarized here, as well as those from previous years, are available on the ICTV website (https://ictv.global/files/proposals/approved). A total of 43 proposals were ratified, leading to the creation of one new phylum, one class, four orders, 33 families, 14 subfamilies, 194 genera and 995 species (Table 1).

**Table 1.** Summary of taxonomic changes for bacterial viruses, ratified in 2025

Taxonomy change	Species	Genus	Subfamily	Family	Order	Class	Phylum
Abolished	22	1	2	–	–	–	–
New	995	194	14	33	3	1	1
Moved or promoted (and/or renamed)	30	120	12	4	1	–	–
Renamed	101	3	–	–	–	–	–

**Fig. 1.**
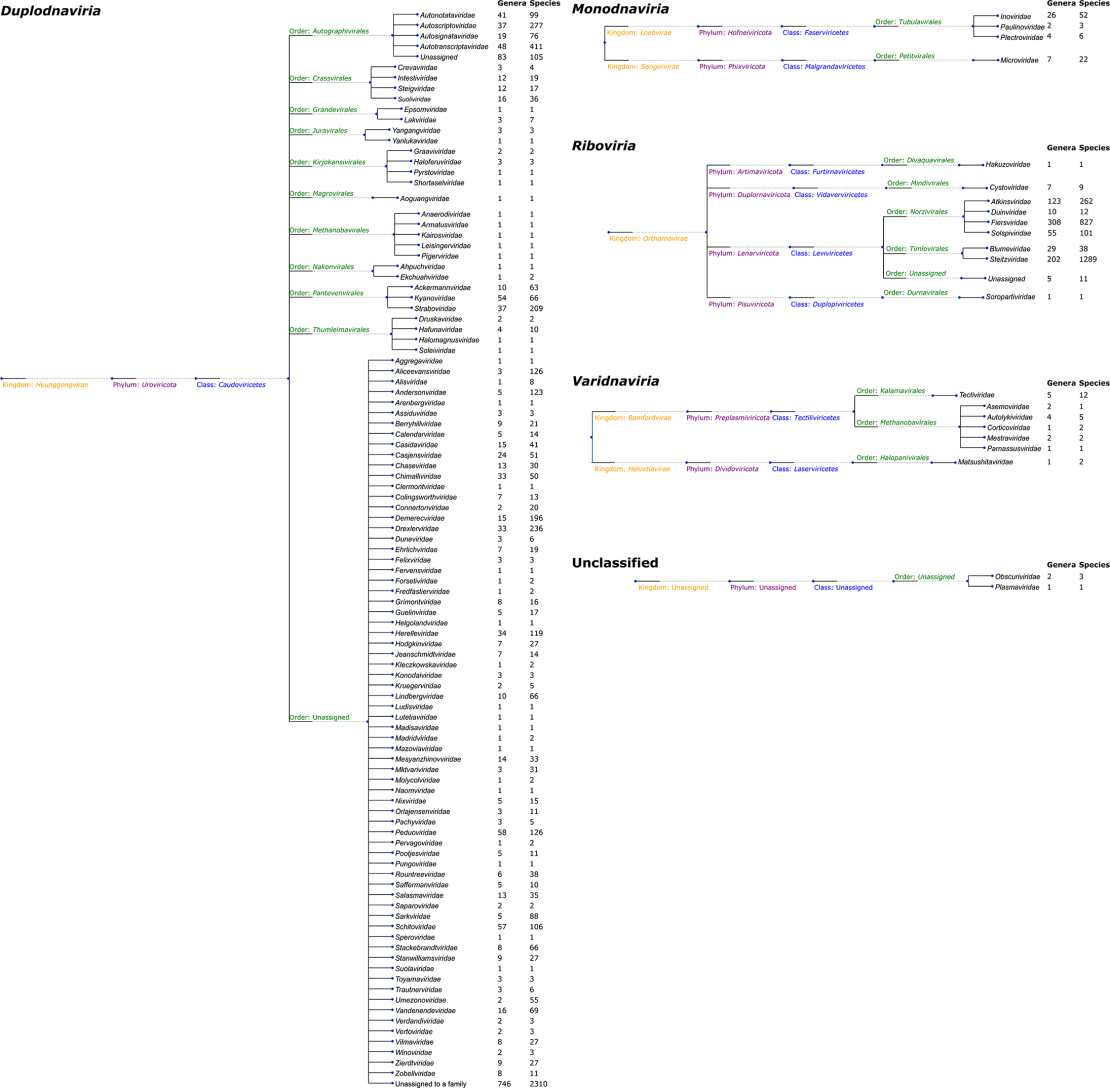
Cladograms depicting the taxonomy of bacterial viruses for the realms *Duplodnaviria*, *Monodnaviria*, *Riboviria*, *Varidnaviria* and the families *Obscuriviridae* and *Plasmaviridae* which have no realm assigned. The columns adjacent to the leaf labels provide counts of the numbers of genera and species by family.

Notable highlights include the creation of the phylum *Artimaviricota* for a group of segmented RNA viruses predicted to infect thermophilic bacteria [8]. The Lak megaphages [9,10] were assigned to the new order *Grandevirales*, while the family *Autographiviridae* was elevated to order (*Autographivirales*), containing four newly defined families. The new order *Pantevenvirales* encompasses the families *Straboviridae*, *Kyanoviridae*, and *Ackermannviridae*. Importantly, the creation of 30 new families continues the process of addressing floating taxa within the class *Caudoviricetes*. This class now contains 31 families assigned to 10 orders, with 70 families remaining unassigned to any order (Fig. 1).

The Bacterial Viruses Subcommittee has also seen growing engagement from the wider research community, as evidenced by an increased number of proposals originating from outside the Subcommittee and its Study Groups. This is encouraged, and we invite researchers to contact the Subcommittee and relevant Study Groups for guidance (https://ictv.global/study-groups?webform_submission_value_1=7). A file including all the Tables of taxonomic changes below is available as a supplementary file to this article.

## Main text

## Contents

2024.001B.Alisviridae_Ludisviridae_Nixviridae_3nf_7ng_24ns
2024.002B.Andersonviridae_1nf_2ng_98ns
2024.003B.Berryhillviridae_1nf_7ng_3mg_10ns
2024.005B.Casidaviridae_1nf_9ng_23ns
2024.006B.Cepavirus_Suseptimavirus_Uetakevirus_1ng_3ns
2024.007B.Chimalliviridae_16mg
2024.008B.Colingsworthviridae_1nf_4ng_3mg_8ns
2024.009B.Connertonviridae_1nf_2mg_12ns
2024.010B.Dovevirinae_1nsf_1ng_12ns
2024.011B.Durnavirales_1nf_1ng_1ns
2024.012B.Ehrlichviridae_1nf_6ng_1mg_9ns
2024.013B.Ferrettivirinae_1nsf_3ng_38ns
2024.014B.Grandevirales_1no_2nf_3nsf_4ng_8ns
2024.015B.Grimontviridae_2ng_1mg_2ns
2024.016B.Hodgkinviridae_1nf_2ng_4mg_5ns
2024.017B.Jeanschmidtviridae_1nf_3ng_4mg_6ns
2024.018B.Kronosvirus_1ng_3ns
2024.019B.Kruegerviridae_1nf_1ng_1mg_4ns
2024.020B.Lindbergviridae_1nf_3ng_7mg_21ns
2024.021B.Malkevirus_1ng_5ns
2024.022B.Markadamsvirinae_1ng_1ns
2024.023B.Mcshanvirinae_1nsf_3ng_25ns
2024.024B.Mtkvariviridae_1nf_1msf_10ns
2024.025B.Obscuriviridae_1nf_2ng_3ns
2024.026B.Pantevenvirales_1no_3mf
2024.028B.Philemonvirus_1ns
2024.029B.Rhodococcus_siphoviruses_7ng_7ns
2024.030B.Trautnerviridae_1nf_1nsf_3ng_6ns
2024.031B.Sarkviridae_1nf_1msf_2mg
2024.032B.Sepahanvirus_1ng_2ns
2024.033B.Mazoviaviridae_1nf_1ng_1ns
2024.034B.Stackebrandtviridae_1nf_2nsf_8mg_8ns
2024.036B.Caudoviricetes_Faserviricetes_Name_Corrections
2024.037B.Vandenendeviridae_1nf_2msf_8ng_1mg_11ns
2024.038B.Vinavirales_3nf_1mf_7ng_5ns_3ms
2024.039B.Artimaviricota_np
2024.040B.Sharonstreetvirus_1ns
2024.041B.Camvirus_2ns
2024.042B.Lacfervirus_1ng_1ns
2024.043B.Cystoviridae_6ng_2nsp_1rng_7rnsp
2024.044B.Felixviridae_1nf_1nsf_2ng_1mg_2ns
2024.045B.Autographivirales

## 2024.001b.Alisviridae_Ludisviridae_NIxviridae_3Nf_7ng_24ns

**Title:** Create three new families (*Alisviridae, Ludisviridae* and *Nixviridae*) with seven new genera and 24 new species

**Authors:** Matrishin CB, Kauffman KM (kmkauffm@buffalo.edu)

**Summary**

**Taxonomic rank(s) affected**
Realm *Duplodnaviria,* kingdom *Heunggongvirae,* phylum *Uroviricota,* class *Caudoviricetes.*
**Description of current taxonomy**
The viruses classified in this proposal do not have a current taxonomic assignment.
**Proposed taxonomic change(s)**
Creation of three new families (*Alisviridae, Ludisviridae* and *Nixviridae*) with seven new genera (*Honmavirus, Ludisvirus, Dewhirstvirus, Nixvirus, Haasevirus*, *Excelsiorvirus* and *Schifferlevirus*) and 24 new species.
**Justification**
A comprehensive analysis of publicly available NCBI *Porphyromonas gingivalis* genomes revealed three new families of viruses, containing seven new genera and 24 new species. This discovery, using a rigorous, complementary bioinformatic approach, revealed what we believe to be precise nucleotide start and end points of the prophage genomes within bacterial contigs. These novel prophages represent the first systematically described phages of *P. gingivalis*. This work, including the proposed taxonomic classifications and figures shown in this proposal, is described in Matrishin *et al*. [11]. Phage genomes are available on NCBI within BioProject PRJNA874424.
**Submitted**: 29 May 24

**Table 2.** *Alisviridae*, 34 new taxa*

**Table:** 

Operation	Rank	New taxon name	Virus name	Exemplar
New taxon	Family	*Alisviridae*		
New taxon	Genus	*Honmavirus*		
New taxon	Species	*Honmavirus pging00B*	Porphyromonas phage phage006a_EM3	BK068089
New taxon	Species	*Honmavirus pging00C*	Porphyromonas phage phage007a_Bg4	PP754929
New taxon	Species	*Honmavirus pging00D*	Porphyromonas phage phage008a_KCOM2795	BK068090
New taxon	Species	*Honmavirus pging00E*	Porphyromonas phage phage010a_HG1691old	PP754930
New taxon	Species	*Honmavirus pging00F*	Porphyromonas phage phage011a_WW2952	BK068092
New taxon	Species	*Honmavirus pging00G*	Porphyromonas phage phage012a_381OKJP	BK068093
New taxon	Species	*Honmavirus pging00H*	Porphyromonas phage phage013a_WW2885	BK068094
New taxon	Species	*Honmavirus pging00I*	Porphyromonas phage phage014a_Kyudai4	BK068095
New taxon	Family	*Ludisviridae*		
New taxon	Genus	*Ludisvirus*		
New taxon	Species	*Ludisvirus pging00A*	Porphyromonas phage phage005b_ATCC49417	PP754928
New taxon	Family	*Nixviridae*		
New taxon	Genus	*Dewhirstvirus*		
New taxon	Species	*Dewhirstvirus pging00J*	Porphyromonas phage phage016a_WW2866	BK068097
New taxon	Species	*Dewhirstvirus pging00K*	Porphyromonas phage phage017a_JCVISC001	BK068098
New taxon	Species	*Dewhirstvirus pging00L*	Porphyromonas phage phage018a_AFR5B1	BK068099
New taxon	Species	*Dewhirstvirus pging00M*	Porphyromonas phage phage019b_ATCC49417	PP754931
New taxon	Genus	*Nixvirus*		
New taxon	Species	*Nixvirus pging00X*	Porphyromonas phage phage032a_KCOM2801	BK068113
New taxon	Genus	*Haasevirus*		
New taxon	Species	*Haasevirus pging00R*	Porphyromonas phage phage025a_SJD11	BK068106
New taxon	Species	*Haasevirus pging00T*	Porphyromonas phage phage027a_F0568	BK068108
New taxon	Species	*Haasevirus pging00U*	Porphyromonas phage phage028a_KCOM2799	BK068109
New taxon	Species	*Haasevirus pging00V*	Porphyromonas phage phage029a_Kyudai3	BK068110
New taxon	Species	*Haasevirus pging00W*	Porphyromonas phage phage030a_KCOM2803	BK068111
New taxon	Genus	*Excelsiorvirus*		
New taxon	Species	*Excelsiorvirus pging00S*	Porphyromonas phage phage026a_KCOM2802	BK068107
New taxon	Genus	*Schifferlevirus*		
New taxon	Species	*Schifferlevirus pging00N*	Porphyromonas phage phage020a_SJD2	BK068101
New taxon	Species	*Schifferlevirus pging00O*	Porphyromonas phage phage022a_WW2931	BK068103
New taxon	Species	*Schifferlevirus pging00P*	Porphyromonas phage phage023a_KCOM2797	BK068104
New taxon	Species	*Schifferlevirus pging00Q*	Porphyromonas phage phage024a_F0570	BK068105

*Source/full text: https://ictv.global/ictv/proposals/2024.001B.Alisviridae_Ludisviridae_Nixviridae_3nf_7ng_24ns.zip.

## 2024.002B.Andersonviridae_1nf_2ng_98ns

**Title:** Create a new family, *Andersonviridae* for the FelixO1-like phages (class: *Caudoviricetes*)

**Authors:** Moraru C, Tolstoy I, Kropinski AM (Phage.Canada@gmail.com)

**Summary**

**Taxonomic rank(s) affected**
Realm *Duplodnaviria,* kingdom *Heunggongvirae,* phylum *Uroviricota,* class *Caudoviricetes.*
**Description of current taxonomy**
At present the following taxa exist as floating genera in the class *Caudoviricetes: Felixounavirus, Kolesnikvirus, Suspvirus* and *Mooglevirus*.
**Proposed taxonomic change(s)**
We propose the creation of one new family, *Andersonviridae*.
To update the genus *Felixounavirus* with 80 new species.
To update the genus *Mooglevirus* with 11 new species.
To add one new species to the genus *Kolesnikvirus.*
To create a new genus *Daniellevirus* with two species.
To create a new genus *Arnovirus* with three species.
**Justification**
We investigated the evolutionary relationships of 123 bacteriophages. Analysis of conserved genes revealed that these phages form a deeply branching monophyletic clade with a distance commensurate with the creation of a new family.
**Submitted**: 01 June 24; **Revised**: 30 September 24

**Table 3.** *Andersonviridae*, 100 new taxa*. Table too large, see supplementary information sheet supp_info_tab_3

**Table 4.** *Andersonviridae*, 1 move taxa*

**Table:** 

Operation	Rank	Taxon name	New parent taxon	Old parent taxon
Move taxon	Subfamily	*Ounavirinae*	*Andersonviridae*	*Caudoviricetes*

**Table 5.** *Andersonviridae*, 2 move; rename taxa*

**Table:** 

Operation	Rank	New taxon name	New parent taxon	Old taxon name
Move; rename taxon	Species	*Mooglevirus susp1*	*Mooglevirus*	*Suspvirus SUSP1*
Move; rename taxon	Species	*Mooglevirus susp2*	*Mooglevirus*	*Suspvirus SUSP2*

**Table 6.** *Andersonviridae*, 1 abolish taxon*

**Table:** 

Operation	Rank	Abolished taxon name
Abolish taxon	Genus	*Suspvirus*

*Source/full text: https://ictv.global/ictv/proposals/2024.002B.Andersonviridae_1nf_2ng_98ns.zip.

## 2024.003B.Berryhillviridae_1nf_7ng_3mg_10ns

**Title:** Create a new family, *Berryhillviridae*, for a group of lytic *Arthrobacter* phages (class: *Caudoviricetes*)

**Authors:** Kurtböke I, Moraru C, Tolstoy I, Kropinski AM (Phage.Canada@gmail.com)

**Summary**

**Taxonomic rank(s) affected**
Realm *Duplodnaviria,* kingdom *Heunggongvirae,* phylum *Uroviricota,* class *Caudoviricetes.*
**Description of current taxonomy**
At present the following taxa exist as floating genera in the class *Caudoviricetes*: genera *Marthavirus, Vibakivirus, Jawnskivirus* and *Ayohtrevirus.*
**Proposed taxonomic change(s)**
We propose the creation of a new family, *Berryhillviridae,* including the existing genera *Marthavirus, Vibakivirus,* and *Ayohtrevirus* in addition to six new genera, *Jinkiesvirus, Jawnskivirus*, *Lilmacvirus, Altadenavirus, Eastwestvirus* and *Sicariusvirus.*
**Justification**
We investigated the evolutionary relationships of 21 bacteriophages. Analysis of conserved genes and tBLASTx distances revealed that these phages form a deeply branching clade at a distance commensurate with the creation of a new family.
**Submitted**: 25 May 24

**Table 7.** *Berryhillviridae*, 18 new taxa*

**Table:** 

Operation	Rank	New taxon name	Virus name	Exemplar
New taxon	Family	*Berryhillviridae*		
New taxon	Genus	*Jawnskivirus*		
New taxon	Species	*Jawnskivirus king2*	Arthrobacter phage King2	MT776811
New taxon	Genus	*Jinkiesvirus*		
New taxon	Species	*Jinkiesvirus jinkies*	Arthrobacter phage Jinkies	MT498043
New taxon	Genus	*Lilmacvirus*		
New taxon	Species	*Lilmacvirus lilmac1015*	Arthrobacter phage Lilmac1015	OL742560
New taxon	Species	*Lilmacvirus bolt007*	Arthrobacter phage Bolt007	OP985600
New taxon	Species	*Lilmacvirus prairie*	Arthrobacter phage Prairie	MW601223
New taxon	Species	*Lilmacvirus klevey*	Arthrobacter phage Klevey	MZ747522
New taxon	Genus	*Altadenavirus*		
New taxon	Species	*Altadenavirus altadena*	Arthrobacter phage Altadena	OR521058
New taxon	Species	*Altadenavirus bumble*	Arthrobacter phage Bumble	MT498055
New taxon	Genus	*Eastwestvirus*		
New taxon	Species	*Eastwestvirus eastwest*	Arthrobacter phage EastWest	OK999980
New taxon	Genus	*Sicariusvirus*		
New taxon	Species	*Sicariusvirus sicarius2*	Arthrobacter phage Sicarius2	MW862982
New taxon	Species	*Sicariusvirus wyborn*	Arthrobacter phage Wyborn	OR475274

**Table 8.** *Berryhillviridae*, 4 move; rename taxa.*

**Table:** 

Operation	Rank	New taxon name	New parent taxon	Old taxon name
Move; rename taxon	Species	*Jawnskivirus jawnski*	*Jawnskivirus*	*Marthavirus jawnski*
Move; rename taxon	Species	*Jawnskivirus beans*	*Jawnskivirus*	*Marthavirus beans*
Move; rename taxon	Species	*Jawnskivirus piccoletto*	*Jawnskivirus*	*Marthavirus piccoletto*
Move; rename taxon	Species	*Jawnskivirus brent*	*Jawnskivirus*	*Marthavirus brent*

**Table 9.** *Berryhillviridae*, 3 move taxa.*

**Table:** 

Operation	Rank	Taxon name	New parent taxon	Old parent taxon
Move taxon	Genus	*Vibakivirus*	*Berryhillviridae*	*Caudoviricetes*
Move taxon	Genus	*Ayohtrevirus*	*Berryhillviridae*	*Caudoviricetes*
Move taxon	Genus	*Marthavirus*	*Berryhillviridae*	*Caudoviricetes*

*Source/full text: https://ictv.global/ictv/proposals/2024.003B.Berryhillviridae_1nf_7ng_3mg_10ns.zip.

## 2024.005B.Casidaviridae_1nf_9ng_23ns

**Title:** Create a new family, *Casidaviridae,* for a group of *Arthrobacter-Microbacterium* phages (class: *Caudoviricetes*)

**Authors:** Kurtböke I, Moraru C, Tolstoy I, Kropinski AM (Phage.Canada@gmail.com)

**Summary**

**Taxonomic rank(s) affected**
Realm *Duplodnaviria,* kingdom *Heunggongvirae,* phylum *Uroviricota,* class *Caudoviricetes.*
**Description of current taxonomy**
At present the following taxa exist as floating genera in the class *Caudoviricetes: Zetavirus, Baileybluvirus, Yangvirus*, *Manhattanvirus* and *Liebevirus.*
**Proposed taxonomic change(s)**
To create a new genus, *Gardenstatevirus,* with two species
To create a new genus, *Percivalvirus,* with two species.
To create a new single species genus *Mabodamacavirus.*
To create a new genus, *Barnstormervirus* with two species.
To create a new single species genus *Honkvirus.*
To create a new single species genus *Cenunavirus.*
To create a new species in *Baileybluvirus.*
To create seven new species in the genus *Yangvirus.*
To create two new species in the genus *Manhattanvirus.*
To create a new single species genus, *Emotionvirus.*
To create a new single species genus, *Hilgardvirus.*
To create a new single species genus, *Swepdovirus.*
To create one new species in the genus *Liebevirus.*
To create a new family, *Casidaviridae.*
**Justification**
We propose the creation of a new family*, Casidaviridae,* after examination of 21 bacteriophages related to the genera *Zetavirus, Baileybluvirus, Yangvirus*, *Manhattanvirus* and *Liebevirus* based on nucleotide sequence similarity, tBLASTx distances and core gene phylogeny.
**Submitted**: 20 May 24; **Revised**: 30 September 24

**Table 10.** *Casidaviridae*, 33 new taxa*

**Table:** 

Operation	Rank	New taxon name	Virus name	Exemplar
New taxon	Family	*Casidaviridae*		
New taxon	Species	*Baileybluvirus callinallbarbz*	Arthrobacter phage CallinAllBarbz	OR553891
New taxon	Species	*Yangvirus janeemi*	Arthrobacter phage Janeemi	ON970616
New taxon	Species	*Yangvirus tuck*	Arthrobacter phage Tuck	OP820474
New taxon	Species	*Yangvirus berrie*	Arthrobacter phage Berrie	PP208921
New taxon	Species	*Yangvirus ascela*	Arthrobacter phage Ascela	OQ709218
New taxon	Species	*Yangvirus cassia*	Arthrobacter phage Cassia	OQ709212
New taxon	Species	*Yangvirus tfortroy*	Arthrobacter phage TforTroy	PP208923
New taxon	Species	*Yangvirus nitro*	Arthrobacter phage Nitro	OR553895
New taxon	Species	*Manhattanvirus vresidence*	Arthrobacter phage VResidence	OP434455
New taxon	Species	*Manhattanvirus wildwest*	Arthrobacter phage Wildwest	OR521060
New taxon	Species	*Liebevirus maguco*	Arthrobacter phage MaGuCo	OQ709203
New taxon	Genus	*Gardenstatevirus*		
New taxon	Species	*Gardenstatevirus gardenstate*	Microbacterium phage GardenState	MT952845
New taxon	Species	*Gardenstatevirus iamgroot*	Microbacterium phage IAmGroot	MK880124
New taxon	Genus	*Percivalvirus*		
New taxon	Species	*Percivalvirus percival*	Microbacterium phage Percival	MH271308
New taxon	Species	*Percivalvirus floof*	Microbacterium phage Floof	MH271298
New taxon	Genus	*Mabodamacavirus*		
New taxon	Species	*Mabodamacavirus mabodamaca*	Microbacterium phage Mabodamaca	OR613467
New taxon	Genus	*Barnstormervirus*		
New taxon	Species	*Barnstormervirus barnstormer*	Microbacterium phage Barnstormer	OQ190478
New taxon	Species	*Barnstormervirus caron*	Microbacterium phage Caron	OQ190481
New taxon	Genus	*Honkvirus*		
New taxon	Species	*Honkvirus honk*	Microbacterium phage Honk	MW862981
New taxon	Genus	*Cenunavirus*		
New taxon	Species	*Cenunavirus Cen1621*	Microbacterium phage Cen1621	ON970568
New taxon	Genus	*Emotionvirus*		
New taxon	Species	*Emotionvirus emotion*	Arthrobacter phage Emotion	OQ709216
New taxon	Genus	*Hilgardvirus*		
New taxon	Species	*Hilgardvirus vroomvroom*	Arthrobacter phage VroomVroom	OQ938592
New taxon	Genus	*Swepdovirus*		
New taxon	Species	*Swepdovirus SWEP2*	Arthrobacter phage SWEP2	ON528933

**Table 11.** *Casidaviridae*, 5 move taxa*

**Table:** 

Operation	Rank	Taxon name	New parent taxon	Old parent taxon
Move taxon	Genus	*Zetavirus*	*Casidaviridae*	*Caudoviricetes*
Move taxon	Genus	*Baileybluvirus*	*Casidaviridae*	*Caudoviricetes*
Move taxon	Genus	*Yangvirus*	*Casidaviridae*	*Azeevirinae*
Move taxon	Genus	*Manhattanvirus*	*Casidaviridae*	*Azeevirinae*
Move taxon	Genus	*Liebevirus*	*Casidaviridae*	*Azeevirinae*

**Table 12.** *Casidaviridae*, 1 abolish taxon*

**Table:** 

Operation	Rank	Abolished taxon name
Abolish taxon	Subfamily	*Azeevirinae*

*Source/full text: https://ictv.global/ictv/proposals/2024.005B.Casidaviridae_1nf_9ng_23ns.zip.

## 2024.006B.Cepavirus_Suseptimavirus_Uetakevirus_1ng_3ns

**Title:** Create a new genus, *Cepavirus,* with one species (*Caudoviricetes; Autographiviridae; Slopekvirinae*) and new species in the genera *Suseptimavirus* (*Caudoviricetes; Gordonclarkvirinae*) and *Uetakevirus* (*Caudoviricetes*).

**Authors:** Pas C, Fieseler L, Briers Y (yves.briers@ugent.be)

**Summary**

**Taxonomic rank(s) affected**
Genus and species.
**Description of current taxonomy**
The bacterial viruses in this proposal are currently unclassified.
**Proposed taxonomic change(s)**
Creation of a new genus, *Cepavirus,* within the subfamily *Slopekvirinae,* family *Autographiviridae*.
Assign Escherichia phage vB_EcoP_PAS7 as a new species in the new genus, *Cepavirus.*
Assign Escherichia phage vB_EcoP_PAS59 as a new species in the genus *Suseptimavirus,* subfamily *Gordonclarkvirinae*.
Assign Escherichia phage vB_EcoP_PAS6 as a new species within the genus *Uetakevirus*.
**Justification**
These bacterial viruses fall within current genus and species demarcation criteria for inclusion within existing genera.
**Submitted:** 27 November 23

**Table 13.** *Cepavirus*, 4 new taxa*

**Table:** 

Operation	Rank	New taxon name	Virus name	Exemplar
New taxon	Genus	*Cepavirus*		
New taxon	Species	*Cepavirus PAS7*	Escherichia phage vB_EcoP_PAS7	OQ921331
New taxon	Species	*Suseptimavirus PAS59*	Escherichia phage vB_EcoP_PAS59	OQ921332
New taxon	Species	*Uetakevirus PAS61*	Escherichia phage vB_EcoP_PAS61	OQ921333

*Source/full text: https://ictv.global/ictv/proposals/2024.006B.Cepavirus_Suseptimavirus_Uetakevirus_1ng_3ns.zip.

## 2024.007B.Chimalliviridae_16mg

**Title:** Move newly classified viral genera into *Chimalliviridae* family and fix previous error

**Author:** Prichard A, Pogliano J (jpogliano@ucsd.edu)

**Summary**

**Taxonomic rank(s) affected**
We propose to move fifteen genera into the *Chimalliviridae* family and remove one genus from this family.
**Description of current taxonomy**
Last year, we submitted a proposal to create a new viral family called *Chimalliviridae* and re-assigned currently classified viruses into this family. This proposal was accepted, but in the meantime, more viruses that belong in this family have been officially recognized and classified by the ICTV. Since these proposals were submitted in the same year as ours, and there was no existing family that was appropriate for them to be assigned to at the time, these viruses were not assigned to any viral families. However, we believe that these viruses belong to the now-recognized *Chimalliviridae* family.
**Proposed taxonomic change(s)**
Members of the newly created genera *Miamivirus, Nimduovirus, Meadowvirus, Branisovskavirus, Ferozepurvirus, Chaoshanvirus, Ludhianavirus, Siatvirus, Maaswegvirus, Eowynvirus, Miltoncavirus, Phabiovirus, Serwervirus, Tepukevirus* and *Pawinskivirus* should be added to the family *Chimalliviridae*. Additionally, the genus *Takahashivirus* should be removed from the family *Chimalliviridae*, as it was included in our proposal last year by mistake and should not belong to this family.
**Justification**
We have redone the phylogenetic analysis used to support the creation of the family *Chimalliviridae* but with the inclusion of several newly classified genera. This shows that these new genera belong in the family *Chimalliviridae,* while *Takahashivirus PBS1,* which we included by mistake, does not.
**Submitted**: 12 June 24

**Table 14.** *Chimalliviridae*, 16 move taxa*

**Table:** 

Operation	Rank	Taxon name	Old parent taxon	New parent taxon
Move taxon	Genus	*Takahashivirus*	*Chimalliviridae*	*Caudoviricetes*
Move taxon	Genus	*Branisovskavirus*	*Caudoviricetes*	*Chimalliviridae*
Move taxon	Genus	*Chaoshanvirus*	*Caudoviricetes*	*Chimalliviridae*
Move taxon	Genus	*Eowynvirus*	*Caudoviricetes*	*Chimalliviridae*
Move taxon	Genus	*Ferozepurvirus*	*Caudoviricetes*	*Chimalliviridae*
Move taxon	Genus	*Ludhianavirus*	*Caudoviricetes*	*Chimalliviridae*
Move taxon	Genus	*Maaswegvirus*	*Caudoviricetes*	*Chimalliviridae*
Move taxon	Genus	*Meadowvirus*	*Caudoviricetes*	*Chimalliviridae*
Move taxon	Genus	*Miamivirus*	*Caudoviricetes*	*Chimalliviridae*
Move taxon	Genus	*Miltoncavirus*	*Caudoviricetes*	*Chimalliviridae*
Move taxon	Genus	*Nimduovirus*	*Caudoviricetes*	*Chimalliviridae*
Move taxon	Genus	*Pawinskivirus*	*Caudoviricetes*	*Chimalliviridae*
Move taxon	Genus	*Phabiovirus*	*Caudoviricetes*	*Chimalliviridae*
Move taxon	Genus	*Serwervirus*	*Caudoviricetes*	*Chimalliviridae*
Move taxon	Genus	*Siatvirus*	*Caudoviricetes*	*Chimalliviridae*
Move taxon	Genus	*Tepukevirus*	*Caudoviricetes*	*Chimalliviridae*

*Source/full text: https://ictv.global/ictv/proposals/2024.007B.Chimalliviridae_16mg.zip.

## 2024.008B.Colingsworthviridae_1nf_4ng_3mg_8ns

**Title:** Create a new family, *Colingsworthviridae,* of *Streptomyces* temperate phages (class *Caudoviricetes*)

**Authors:** Kurtböke I, Moraru C, Tolstoy I, Kropinski AM (Phage.Canada@gmail.com)

**Summary**

**Taxonomic rank(s) affected**
Realm *Duplodnaviria,* kingdom *Heunggongvirae,* phylum *Uroviricota,* class *Caudoviricetes.*
**Description of current taxonomy**
Three taxa of temperate *Streptomyces* phages exist as floating genera in the class *Caudoviricetes,* namely, *Vashvirus*, *Tigunavirus* and *Lomovskayavirus*. Up to the present, no effort has been made to assign them to higher taxa.
**Proposed taxonomic change(s)**
Create a new single species genus *Shadyvirus.*
Create a new single species genus *Sycamorevirus.*
Create a new single species genus *Shaekyvirus.*
Create a new genus *Sebastisaurusvirus* with three species.
To add a single new species to the genus *Vashvirus.*
To add a single new species to the genus *Lomovskayavirus.*
To create a new family, *Colingsworthviridae,* for these genera.
To transfer *Vashvirus, Tigunavirus* and *Lomovskayavirus* to this new family.
**Justification**
As a result of detailed genomic, proteomic and phylogenetic analyses using VIRIDIC, ViPTree, VirClust, we propose to create four new genera of Phi-C31-like temperate siphoviruses, in a new family named in honour of the first person to isolate a *Streptomyces* bacteriophage, Dr. Donald Colingsworth.
**Submitted**: 15 May 24; **Revised**: 30 September 24

**Table 15.** *Colingsworthviridae*, 13 new taxa*

**Table:** 

Operation	Rank	New taxon name	Virus name	Exemplar
New taxon	Family	*Colingsworthviridae*		
New taxon	Genus	*Shadyvirus*		
New taxon	Species	*Shadyvirus shady*	Streptomyces phage Shady	MT701596
New taxon	Genus	*Sycamorevirus*		
New taxon	Species	*Sycamorevirus sycamore*	Streptomyces phage Sycamore	MT701593
New taxon	Genus	*Shaekyvirus*		
New taxon	Species	*Shaekyvirus shaeky*	Streptomyces phage Shaeky	MT701595
New taxon	Genus	*Sebastisaurusvirus*		
New taxon	Species	*Sebastisaurusvirus sebastisaurus*	Streptomyces phage Sebastisaurus	MK450433
New taxon	Species	*Sebastisaurusvirus heather*	Streptomyces phage Heather	MK686069
New taxon	Species	*Sebastisaurusvirus remusloopin*	Streptomyces phage RemusLoopin	MK686068
New taxon	Species	*Vashvirus euratis*	Streptomyces phage Euratis	MK450426
New taxon	Species	*Lomovskayavirus shawty*	Streptomyces phage Shawty	MK433266

**Table 16.** *Colingsworthviridae*, 3 move taxa*

**Table:** 

Operation	Rank	Taxon name	New parent taxon	Old parent taxon
Move taxon	Genus	*Vashvirus*	*Colingsworthviridae*	*Caudoviricetes*
Move taxon	Genus	*Lomovskayavirus*	*Colingsworthviridae*	*Caudoviricetes*
Move taxon	Genus	*Tigunavirus*	*Colingsworthviridae*	*Caudoviricetes*

*Source/full text: https://ictv.global/ictv/proposals/2024.008B.Colingsworthviridae_1nf_4ng_3mg_8ns.zip.

## 2024.009B.Connertonviridae_1nf_2mg_12ns

**Title:** Create a new family, *Connertonviridae* for a group of *Campylobacter* phages (class: *Caudoviricetes*)

**Authors:** Moraru C, Tolstoy I, Kropinski AM (Phage.Canada@gmail.com)

**Summary**

**Taxonomic rank(s) affected**
Realm *Duplodnaviria,* kingdom *Heunggongvirae,* phylum *Uroviricota,* class *Caudoviricetes.*
**Description of current taxonomy**
At present the following taxa exist as genera within the floating subfamily *Eucampyvirinae,* class *Caudoviricetes: Fletchervirus* and *Firehammervirus*.
**Proposed taxonomic change(s)**
To create eight new species in the genus *Fletchervirus.*
To create four new species in the genus *Firehammervirus.*
To create a new family *Connertonviridae* and abolish the subfamily *Eucampyvirinae*.
To move the genera *Fletchervirus* and *Firehammervirus* into this family.
**Justification**
We propose the abolishment of the subfamily *Eucampyvirinae* and the creation of a new family *Connertonviridae* based on analysis of the genera *Fletchervirus* and *Firehammervirus* using VIRIDIC, ViPTree, VirClust and phylogeny of 16 core proteins shared between the member species.
**Submitted**: 30 May 24

**Table 17.** *Connertonviridae*, 13 new taxa*

**Table:** 

Operation	Rank	New taxon name	Virus name	Exemplar
New taxon	Family	*Connertonviridae*		
New taxon	Species	*Fletchervirus F341*	Campylobacter phage F341	OQ864999
New taxon	Species	*Fletchervirus QDYZ*	Campylobacter phage vB_Cj_QDYZ	OQ515481
New taxon	Species	*Fletchervirus PC5*	Campylobacter phage PC5	KX229736
New taxon	Species	*Fletchervirus F336*	Campylobacter phage F336	MT863715
New taxon	Species	*Fletchervirus CJLB7*	Campylobacter phage CJLB-7	MW057933
New taxon	Species	*Fletchervirus CJLB10*	Campylobacter phage CJLB-10	MW074124
New taxon	Species	*Fletchervirus F372*	Campylobacter phage F372	MT863729
New taxon	Species	*Fletchervirus F207*	Campylobacter phage F207	MT863714
New taxon	Species	*Firehammervirus CJLB15*	Campylobacter phage CJLB-15	MW365733
New taxon	Species	*Firehammervirus F379*	Campylobacter phage F379	MT932329
New taxon	Species	*Firehammervirus CJLB12*	Campylobacter phage CJLB-12	MW074125
New taxon	Species	*Firehammervirus CJLB14*	Campylobacter phage CJLB-14	MW074126

**Table 18.** *Connertonviridae*, 2 move taxa*

**Table:** 

Operation	Rank	Taxon name	New parent taxon	Old parent taxon
Move taxon	Genus	*Fletchervirus*	*Connertonviridae*	*Eucampyvirinae*
Move taxon	Genus	*Firehammervirus*	*Connertonviridae*	*Eucampyvirinae*

**Table 19.** *Connertonviridae*, 1 abolish taxon*

**Table:** 

Operation	Rank	Abolished taxon name
Abolish taxon	Subfamily	*Eucampyvirinae*

*Source/full text: https://ictv.global/ictv/proposals/2024.009B.Connertonviridae_1nf_2mg_12ns.zip.

## 2024.010B.Dovevirinae_1nsf_1ng_12ns

**Title:** Create a new subfamily, *Dovevirinae,* with two genera for a group of lytic *Gordonia* phages (class: *Caudoviricetes*)

**Authors:** Kurtböke I, Moraru C, Tolstoy I, Kropinski AM (Phage.Canada@gmail.com)

**Summary**

**Taxonomic rank(s) affected**
Realm *Duplodnaviria,* kingdom *Heunggongvirae,* phylum *Uroviricota,* class *Caudoviricetes.*
**Description of current taxonomy**
Currently phages grouped within cluster DV in the Actinobacteriophage Database are recognized in the genus *Lambovirus*. These are lytic siphophages with circularly permuted genomes infecting *Gordonia* species.
**Proposed taxonomic change(s)**
The creation of a new subfamily, *Dovevirinae,* including two genera, *Lambovirus* and *Xeniaduovirus,* is proposed.
**Justification**
Constituent species in these genera exhibit greater than 50% nucleotide sequence similarity supporting their assignment to a new subfamily.
**Submitted**: 01 June 24

**Table 20.** *Dovevirinae*, 14 new taxa*

**Table:** 

Operation	Rank	New taxon name	Virus name	Exemplar
New taxon	Subfamily	*Dovevirinae*		
New taxon	Genus	*Xeniaduovirus*		
New taxon	Species	*Xeniaduovirus xenia2*	Gordonia phage Xenia2	PP725409
New taxon	Species	*Lambovirus wojtek*	Gordonia phage Wojtek	OL455890
New taxon	Species	*Lambovirus genamy16*	Gordonia phage Genamy16	ON755185
New taxon	Species	*Lambovirus novasharks*	Gordonia phage NovaSharks	ON755187
New taxon	Species	*Lambovirus zany*	Gordonia phage Zany	OL455887
New taxon	Species	*Lambovirus erutan*	Gordonia phage Erutan	OR475273
New taxon	Species	*Lambovirus jalebi*	Gordonia phage Jalebi	OL455895
New taxon	Species	*Lambovirus birthdayboy*	Gordonia phage BirthdayBoy	OR475261
New taxon	Species	*Lambovirus fulcrum*	Gordonia phage Fulcrum	OR521071
New taxon	Species	*Lambovirus parvustarda*	Gordonia phage ParvusTarda	OP172868
New taxon	Species	*Lambovirus otterstedtS21*	Gordonia phage OtterstedtS21	OP172870
New taxon	Species	*Lambovirus patos*	Gordonia phage Patos	OP172876

**Table 21.** *Dovevirinae*, 1 move taxon*

**Table:** 

Operation	Rank	Taxon name	New parent taxon	Old parent taxon
Move taxon	Genus	*Lambovirus*	*Dovevirinae*	*Caudoviricetes*

*Source/full text: https://ictv.global/ictv/proposals/2024.010B.Dovevirinae_1nsf_1ng_12ns.zip.

## 2024.011B.Durnavirales_1nf_1ng_1ns

**Title:** Create a new family, *Soropartiviridae,* within the order *Durnavirales* for classification of partiti-like virus infecting thermoacidophilic bacteria

**Authors:** Syun-ichi Urayama (urayama.shunichi.gn@u.tsukuba.ac.jp), Akihito Fukudome, Eugene V. Koonin, Takuro Nunoura, Mart Krupovic (mart.krupovic@pasteur.fr)

**Summary**

**Taxonomic rank(s) affected**
*Riboviria, Orthornavirae, Pisuviricota, Duplopiviricetes, Durnavirales.*
**Description of current taxonomy**
Order *Durnavirales* includes six families of viruses with double-stranded RNA genomes. Most of the durnavirals infect fungal hosts, with the exception of partitivirids, which besides fungi, infect plants and protozoa, and picobirnavirids, which appear to infect bacteria.
**Proposed taxonomic change(s)**
Create a new family, *Soropartiviridae,* with a genus, *Caliparnavirus,* within the order *Durnavirales* to classify partiti-like viruses discovered in the hot spring samples and infecting thermoacidophilic bacteria.
**Justification**
Phylogenetic analysis based on the RNA-directed RNA polymerase (RdRP) placed the new group of bacterial partiti-like viruses outside of the established *Partitiviridae* genera. Furthermore, unlike all other classified partivirids, one of the two segments of soropartivirids is bicistronic.
**Submitted**: 21 June 24

**Table 22.** *Durnavirales*, 3 new taxa*

**Table:** 

Operation	Rank	New taxon name	Virus name	Exemplar
New taxon	Family	*Soropartiviridae*		
New taxon	Genus	*Caliparnavirus*		
New taxon	Species	*Caliparnavirus acidus*	hot spring partiti-like virus 1	RNA1: BTCP01000001; RNA2: BTCP01000004

*Source/full text: https://ictv.global/ictv/proposals/2024.011B.Durnavirales_1nf_1ng_1ns.zip.

## 2024.012B.Ehrlichviridae_1nf_6ng_1mg_9ns

**Title:** Create a new family, *Ehrlichviridae,* for a group of *Bacillus* Andromeda-like phages (class: *Caudoviricetes*)

**Authors:** Barylski J (b54026@amu.edu.pl), Moraru C, Tolstoy I, Kropinski AM

**Summary**

**Taxonomic rank(s) affected**
Realm *Duplodnaviria,* kingdom *Heunggongvirae,* phylum *Uroviricota,* class *Caudoviricetes.*
**Description of current taxonomy**
The genus *Andromedavirus* currently exists as a floating genus in the class *Caudoviricetes.*
**Proposed taxonomic change(s)**
To create a new genus *Suttonboningtonvirus* with one species.
To create a new genus *Gettysburgvirus* with three species.
To add three new species to the genus *Andromedavirus.*
To create a new single species genus *Anathvirus.*
To create a new single species genus *Dazunavirus.*
To create a new single species genus *Chennaivirus.*
To create a new single species genus *Nairobivirus.*
To create a new family, *Ehrlichviridae,* for the above-mentioned taxa.
**Justification**
The phages comprising these taxa form a deep branching clade using tBLASTx distances and single gene phylogeny. Core gene analysis shows the presence of 15 proteins conserved across all members of the proposed family.
**Submitted**: 19 April 24

**Table 23.** *Ehrlichviridae*, 16 new taxa*

**Table:** 

Operation	Rank	New taxon name	Virus name	Exemplar
New taxon	Family	*Ehrlichviridae*		
New taxon	Species	*Andromedavirus leo2*	Bacillus phage Leo2	KU836751
New taxon	Genus	*Suttonboningtonvirus*		
New taxon	Species	*Suttonboningtonvirus sv1ICo2020*	Bacillus phage 1_ICo-2020	MT700412
New taxon	Genus	*Gettysburgvirus*		
New taxon	Species	*Gettysburgvirus gv056SW001B*	Bacillus phage 056SW001B	MN176230
New taxon	Species	*Gettysburgvirus gv268TH004*	Bacillus phage 268TH004	MW394467
New taxon	Species	*Gettysburgvirus gv019DV002*	Bacillus phage 019DV002	MN176220
New taxon	Genus	*Anathvirus*		
New taxon	Species	*Anathvirus anath*	Bacillus phage Anath	MG983742
New taxon	Genus	*Dazunavirus*		
New taxon	Species	*Dazunavirus DZ1*	Bacillus phage DZ1	OR338916
New taxon	Genus	*Chennaivirus*		
New taxon	Species	*Chennaivirus MVCVPHSA1*	Staphylococcus phage MVC_VPHSA1	OR670591
New taxon	Genus	*Nairobivirus*		
New taxon	Species	*Nairobivirus nv36*	Bacillus phage vB_BpsS-36	MH884513

**Table 24.** *Ehrlichviridae*, 1 move taxon*

**Table:** 

Operation	Rank	Taxon name	New parent taxon	Old parent taxon
Move taxon	Genus	*Andromedavirus*	*Ehrlichviridae*	*Caudoviricetes*

*Source/full text: https://ictv.global/ictv/proposals/2024.012B.Ehrlichviridae_1nf_6ng_1mg_9ns.zip.

## 2024.013B.Ferrettivirinae_1nsf_3ng_38ns

**Title:** To create a new subfamily, *Ferrettivirinae,* for *Streptococcus* prophages (class: *Caudoviricetes*)

**Authors:** Tolstoy I, Moraru C, Kropinski AM (Phage.Canada@gmail.com)

**Summary**

**Taxonomic rank(s) affected**
Realm *Duplodnaviria,* kingdom *Heunggongvirae,* phylum *Uroviricota,* class *Caudoviricetes.*
**Description of current taxonomy**
The viruses classified in this proposal do not have a current taxonomic assignment.
**Proposed taxonomic change(s)**
We propose a new subfamily in the class *Caudoviricetes,* named in honour of Professor Joseph J. Ferretti, of *Streptococcus* temperate siphoprophages including three genera: *Spinunavirus, Norfolkplacevirus* and *Hinxtonvirus*.
**Justification**
Collectively, these phages share ≥29.7% DNA sequence similarity and 14 homologous proteins (22.2% in common).
**Submitted**: 07 May 24

**Table 25.** *Ferrettivirinae*, 42 new taxa*. Table too large, see supplementary information sheet supp_info_tab_25

*Source/full text: https://ictv.global/ictv/proposals/2024.013B.Ferrettivirinae_1nsf_3ng_38ns.zip

## 2024.014B.Grandevirales_1no_2nf_3nsf_4ng_8ns

**Title:** Create one new order *Grandevirales* (*Duplodnaviria*)

**Authors:** Cook R, Pye HV, Crisci MA, Telatin A, Santini JM (j.santini@ucl.ac.uk), Adriaenssens EM (Evelien.adriaenssens@quadram.ac.uk)

**Summary**

**Taxonomic rank(s) affected**
Realm *Duplodnaviria,* kingdom *Heunggongvirae,* phylum *Uroviricota,* class *Caudoviricetes.*
**Description of current taxonomy**
The viruses classified in this proposal do not have a current taxonomic assignment.
**Proposed taxonomic change(s)**
We propose the creation of one new order, *Grandevirales,* including two new families, *Lakviridae* and *Epsomviridae,* three sub-families (*Quingentivirinae, Quadringentisvirinae* and *Sescentorumvirinae)* and four genera (*Vetruanivirus, Hatfieldvirus, Amboselivirus* and *Wendovervirus*). We also propose the creation of eight novel species within these genera.
**Justification**
We investigated the evolutionary relationships of 23 megaphage genomes with sizes greater than 400 kb and propose a taxonomy for their classification. Analysis of their putative proteins revealed that Lak phages form a deeply branching monophyletic clade within the class *Caudoviricetes* apart from all other genomes, and hence justifies the creation of a new order *Grandevirales*. One of the interesting features of this clade is that all current members are characterized by an alternative genetic code, where the TAG stop codon is repurposed to encode an amino acid.
**Submitted**: 04 June 23; **Revised**: 07 October 24

**Table 26** *Grandevirales*, 18 new taxa*

**Table:** 

Operation	Rank	New taxon name	Virus name	Exemplar
New taxon	Order	*Grandevirales*		
New taxon	Family	*Lakviridae*		
New taxon	Subfamily	*Quingentivirinae*		
New taxon	Genus	*Vetruanivirus*		
New taxon	Species	*Vetruanivirus primi*	Prevotella phage Lak-A1	MK250015
New taxon	Species	*Vetruanivirus secundi*	Prevotella phage Lak-A2	MK250019
New taxon	Species	*Vetruanivirus dhakaense*	Prevotella phage Lak-C1	MK250029
New taxon	Species	*Vetruanivirus porcinprimi*	uncultured phage RVC AP1_GC26	OR769218
New taxon	Species	*Vetruanivirus porcinsecundi*	uncultured phage RVC AP3_GC26	OR769219
New taxon	Genus	*Amboselivirus*		
New taxon	Species	*Amboselivirus simi*	Prevotella phage Lak-B1	MK250020
New taxon	Subfamily	*Quadringentisvirinae*		
New taxon	Genus	*Hatfieldvirus*		
New taxon	Species	*Hatfieldvirus porci*	uncultured phage RVC_JS4_GC31	OR769222
New taxon	Family	*Epsomviridae*		
New taxon	Subfamily	*Sescentorumvirinae*		
New taxon	Genus	*Wendovervirus*		
New taxon	Species	*Wendovervirus sonii*	uncultured phage HB1	OR769223

*Source/full text: https://ictv.global/ictv/proposals/2024.014B.Grandevirales_1no_2nf_3nsf_4ng_8ns.zip.

## 2024.015B.Grimontviridae_2ng_1mg_2ns

**Title:** To update the family *Grimontviridae* through the addition of three genera (class: *Caudoviricetes*)

**Authors:** Dechesne A, Moraru C, Parra B, Tolstoy I, Kropinski AM (Phage.Canada@gmail.com)

**Summary**

**Taxonomic rank(s) affected**
Realm *Duplodnaviria,* kingdom *Heunggongvirae,* phylum *Uroviricota,* class *Caudoviricetes,* family *Grimontviridae.*
**Description of current taxonomy**
The C3 group of podoviruses are a very rare morphotype possessing elongated capsids, the ‘type virus’ of which is Escherichia phage phiEco32. The family *Grimontviridae* currently consists of five genera: *Crifsvirus, Dalianvirus, Libingvirus*, *Moazamivirus* and *Privateervirus*.
**Proposed taxonomic change(s)**
Create a new single species genus *Lundtoftevirus* Transfer the genus *Lahexavirus* to the family *Grimontviridae.*
**Justification**
The family is represented by a cohesive and monophyletic group in the main predicted proteome-based clustering tools (VirClust, ViPTree and vConTACT2). Members of the family share 12 core proteins.
**Submitted**: 06 May 24; **Revised**: 30 June 24

**Table 27** *Grimontviridae*, 2 new taxa.*

**Table:** 

Operation	Rank	New taxon name	Virus name	Exemplar
New taxon	Genus	*Lundtoftevirus*		
New taxon	Species	*Lundtoftevirus Lu221*	IncN phage Lu221	OQ829281

**Table 28** *Grimontviridae*, 2 move taxa*

**Table:** 

Operation	Rank	Taxon name	New parent taxon	Old parent taxon
Move taxon	Genus	*Lahexavirus*	*Grimontviridae*	*Caudoviricetes*
Move taxon	Genus	*Aquaneticvirus*	*Grimontviridae*	*Caudoviricetes*

*Source/full text: https://ictv.global/ictv/proposals/2024.015B.Grimontviridae_2ng_1mg_2ns.zip.

## 2024.016B.Hodgkinviridae_1nf_2ng_4mg_5ns

**Title:** Create a new family, *Hodgkinviridae,* for a group of lytic *Microbacterium* phages (class: *Caudoviricetes*)

**Authors:** Kurtböke I, Moraru C, Tolstoy I, Kropinski AM (Phage.Canada@gmail.com)

**Summary**

**Taxonomic rank(s) affected**
Realm *Duplodnaviria,* kingdom *Heunggongvirae,* phylum *Uroviricota,* class *Caudoviricetes.*
**Description of current taxonomy**
The genera *Mementomorivirus, Quhwahvirus* and *Meganvirus* exist as floating genera in the class *Caudoviricetes.*
**Proposed taxonomic change(s)**
To create a new single-species genus, *Fuzzbustervirus*
To add a single new species to the genus *Kozievirus.*
To split the genus *Mementomorivirus* in two, creating *Margaeryvirus.*
To add a single new species to the genus *Meganvirus.*
To add two species to the genus *Quhwahvirus.*
To create a new family, *Hodgkinviridae,* for these genera and *Metamorphoovirus.*
**Justification**
Using VIRIDIC, ViPTree, VIRCLUST and vConTACT v.3.0, we have established that this is a cohesive group of lytic *Microbacterium* siphoviruses which share ≥12.2% DNA sequence similarity and 14 common proteins.
**Submitted**: 27 May 24; **Revised**: 30 September 24

**Table 29** *Hodgkinviridae*, 8 new taxa*

**Table:** 

Operation	Rank	New taxon name	Virus name	Exemplar
New taxon	Family	*Hodgkinviridae*		
New taxon	Species	*Kozievirus MO526*	Microbacterium phage MO526	OR941552
New taxon	Species	*Meganvirus nichole72*	Microbacterium phage Nicole72	OR159674
New taxon	Species	*Quhwahvirus pulchra*	Microbacterium phage Pulchra	MW601217
New taxon	Species	*Quhwahvirus littlefortune*	Microbacterium phage LittleFortune	OR475280
New taxon	Genus	*Fuzzbustervirus*		
New taxon	Species	*Fuzzbustervirus fuzzbuster*	Microbacterium phage FuzzBuster	MN062720
New taxon	Genus	*Margaeryvirus*		

**Table 30** *Hodgkinviridae*, 4 move taxa*

**Table:** 

Operation	Rank	Taxon name	New parent taxon	Old parent taxa
Move taxon	Subfamily	*Kutznervirinae*	*Hodgkinviridae*	*Caudoviricetes*
Move taxon	Genus	*Meganvirus*	*Hodgkinviridae*	*Kutznervirinae*
Move taxon	Genus	*Quhwahvirus*	*Hodgkinviridae*	*Kutznervirinae*
Move taxon	Genus	*Metamorphoovirus*	*Hodgkinviridae*	*Kutznervirinae*

**Table 31** *Hodgkinviridae*, 2 move; rename taxa*

**Table:** 

Operation	Rank	New taxon name	New parent taxon	Old taxon name
Move; rename taxon	Species	*Margaeryvirus margaery*	*Margaeryvirus*	*Mementomorivirus margaery*
Move; rename taxon	Species	*Margaeryvirus terij*	*Margaeryvirus*	*Mementomorivirus terij*

*Source/full text: https://ictv.global/ictv/proposals/2024.016B.Hodgkinviridae_1nf_2ng_4mg_5ns.zip.

## 2024.017B.Jeanschmidtviridae_1nf_3ng_4mg_6ns

**Title:** Create a new family, *Jeanschmidtviridae* for a group of *Caulobacter* and *Brevundimonas* phages (class: *Caudoviricetes*)

**Authors:** Millard A, Moraru C, Tolstoy I, Kropinski AM (Phage.Canada@gmail.com)

**Summary**

**Taxonomic rank(s) affected**
Realm *Duplodnaviria,* kingdom *Heunggongvirae,* phylum *Uroviricota,* class *Caudoviricetes.*
**Description of current taxonomy**
The taxa *Colossusvirus, Bertelyvirus*, *Shapirovirus* and *Poindextervirus* are floating genera in the class *Caudoviricetes.*
**Proposed taxonomic change(s)**
To create a new genus, *Kikimoravirus,* with two species.
To create a new genus, *Marchewkavirus,* with three species.
To create a single-species genus, *Bajunvirus.* Abolish the subfamily *Dolichocephalovirinae.*
To create a new family, *Jeanschmidtviridae,* for these genera and *Colossusvirus, Bertelyvirus*, *Shapirovirus* and *Poindextervirus*.
**Justification**
The proposed members share ≥10.3% DNA sequence similarity and share 38 protein homologs.
**Submitted**: 10 June 24; **Revised**: 30 September 24

**Table 32** *Jeanschmidtviridae*, 10 new taxa*

**Table:** 

Operation	Rank	New taxon name	Virus name	Exemplar
New taxon	Family	*Jeanschmidtviridae*		
New taxon	Genus	*Kikimoravirus*		
New taxon	Species	*Kikimoravirus kikimora*	Brevundimonas phage vB_BpoS-Kikimora	ON529857
New taxon	Species	*Kikimoravirus gurke*	Brevundimonas phage vB_BpoS-Gurke	ON529850
New taxon	Genus	*Marchewkavirus*		
New taxon	Species	*Marchewkavirus marchewka*	Brevundimonas phage vB_BpoS-Marchewka	ON529851
New taxon	Species	*Marchewkavirus kabachok*	Brevundimonas phage vB_BpoS-Kabachok	ON529852
New taxon	Species	*Marchewkavirus domovoi*	Brevundimonas phage vB_BpoS-Domovoi	ON529855
New taxon	Genus	*Bajunvirus*		
New taxon	Species	*Bajunvirus bajun*	Brevundimonas phage vB_BpoS-Bajun	ON529858

**Table 33.** *Jeanschmidtviridae*, 4 move taxa*

**Table:** 

Operation	Rank	Taxon name	New parent taxon	Old parent taxon
Move taxon	Genus	*Colossusvirus*	*Jeanschmidtviridae*	*Dolichocephalovirinae*
Move taxon	Genus	*Bertelyvirus*	*Jeanschmidtviridae*	*Dolichocephalovirinae*
Move taxon	Genus	*Shapirovirus*	*Jeanschmidtviridae*	*Dolichocephalovirinae*
Move taxon	Genus	*Poindextervirus*	*Jeanschmidtviridae*	*Dolichocephalovirinae*

**Table 34.** *Jeanschmidtviridae*, 1 abolish taxon*

**Table:** 

Operation	Rank	Abolished taxon name
Abolish taxon	Subfamily	*Dolichocephalovirinae*

*Source/full text: https://ictv.global/ictv/proposals/2024.017B.Jeanschmidtviridae_1nf_3ng_4mg_6ns.zip.

## 2024.018B.Kronosvirus_1ng_3ns

**Title:** Create one new genus (*Kronosvirus*) with three species (*Caudoviricetes*).

**Author:** Ely B (ely@sc.edu)

**Summary**

**Taxonomic rank(s) affected**
Realm *Duplodnaviria,* kingdom *Heunggongvirae,* phylum *Uroviricota,* class *Caudoviricetes.*
**Description of current taxonomy**
The viruses classified in this proposal do not have a current taxonomic assignment.
**Proposed taxonomic change(s)**
Caulobacter bacteriophage Kronos is a unique siphovirus with a 50 nm icosahedral head and a long (150 nm) flexible tail. Aside from two phages, there are no close matches of its genome sequence to any other bacteriophage in the GenBank database. Additional phages that are closely related to Kronos can be grouped into three distinct species based on the ICTV criteria.
**Justification**
The creation of the genus *Kronosvirus* complies with current demarcation criteria [2].
**Submitted**: 27 May 24

**Table 35.** *Kronosvirus*, 4 new taxa*

**Table:** 

Operation	Rank	New taxon name	Virus name	Exemplar
New taxon	Genus	*Kronosvirus*		
New taxon	Species	*Kronosvirus pelion*	Caulobacter phage Kronos	MH884648
New taxon	Species	*Kronosvirus pomeria*	Caulobacter phage TMCBR2	OQ269668
New taxon	Species	*Kronosvirus elgin*	Caulobacter phage TMCBR4	OQ330850

*Source/full text: https://ictv.global/ictv/proposals/2024.018B.Kronosvirus_1ng_3ns.zip.

## 2024.019B.Kruegerviridae_1nf_1ng_1mg_4ns

**Title:** Create a new family, *Kruegerviridae,* for a group of *Gordonia* phages (class: *Caudoviricetes*)

**Authors:** Kurtböke I, Moraru C, Tolstoy I, Kropinski AM (Phage.Canada@gmail.com)

**Summary**

**Taxonomic rank(s) affected**
Realm *Duplodnaviria,* kingdom *Heunggongvirae,* phylum *Uroviricota,* class *Caudoviricetes.*
**Description of current taxonomy**
*Vanleevirus* currently exists as a floating genus in the class *Caudoviricetes.*
**Proposed taxonomic change(s)**
To create a new genus *Cafassovirus* with four species.
To create a new family, *Kruegerviridae,* for the genera *Cafassovirus* and *Vanleevirus*.
**Justification**
By VIRIDIC analysis, members of these two genera share ≥18.8% DNA sequence similarity and also share 46 protein homologs. The genera *Vanleevirus* and *Cafassovirus* form a deep-branching clade using tBLASTx distances, commensurate with the establishment of a new family of bacterial viruses.
**Submitted**: 28 May 24; **Revised**: 30 September 24

**Table 36.** *Kruegerviridae*, 6 new taxa*

**Table:** 

Operation	Rank	New taxon name	Virus name	Exemplar
New taxon	Family	*Kruegerviridae*		
New taxon	Genus	*Cafassovirus*		
New taxon	Species	*Cafassovirus cafasso*	Gordonia phage Cafasso	MZ322021
New taxon	Species	*Cafassovirus morgana*	Gordonia phage Morgana	PP537962
New taxon	Species	*Cafassovirus aleemily*	Gordonia phage Aleemily	ON970578
New taxon	Species	*Cafassovirus obladi*	Gordonia phage ObLaDi	OP297535

**Table 37.** *Kruegerviridae*, 1 move taxon*

**Table:** 

Operation	Rank	Taxon name	New parent taxon	Old parent taxon
Move taxon	Genus	*Vanleevirus*	*Kruegerviridae*	*Caudoviricetes*

*Source/full text: https://ictv.global/ictv/proposals/2024.019B.Kruegerviridae_1nf_1ng_1mg_4ns.zip.

## 2024.020B.Lindbergviridae_1nf_3ng_7mg_21ns

**Title:** Create a new family, *Lindbergviridae,* for PB1-like phages (class: *Caudoviricetes*)

**Authors:** Moraru C, Tolstoy I, Kropinski AM (Phage.Canada@gmail.com)

**Summary**

**Taxonomic rank(s) affected**
Realm *Duplodnaviria,* kingdom *Heunggongvirae,* phylum *Uroviricota,* class *Caudoviricetes.*
**Description of current taxonomy**
PB1-like phages have been classified into the following genera: *Kylevirus, Tabernariusvirus, Bcepfunavirus, Pbunavirus, Wifcevirus*, *Myosmarvirus* and *Carpasinavirus*. All these are myoviruses infecting members of the Betaproteobacteria and Gammaproteobacteria.
**Proposed taxonomic change(s)**
To create ten new species in the genus *Pbunavirus.*
To create one new species in the genus *Myosmarvirus.*
To add six new species to the genus *Wifcevirus.*
To add one new species to the genus *Carpasinavirus.*
To create a new single species genus *Gladiolivirus.*
To create a new single species genus *Irusalimvirus.*
To create a new single species genus *Plutovirus.*
To create a new family, *Lindbergviridae,* for the above-mentioned taxa as well as *Kylevirus, Tabernariusvirus* and *Bcepfunavirus*.
**Justification**
All our genomic and proteomic analyses reveal that the previously established genera *Kylevirus* (2020.086B.R.Kylevirus), *Tabernariusvirus* (2018.099B.A.v1.Tabernariusvirus), *Bcepfunavirus* (2020.116B.R.Pbunavirus), *Pbunavirus, Wifcevirus, Myosmarvirus* and *Carpasinavirus* together with the three new genera listed above belong to a new family which we have named in honour of Alf A. Lindberg. The bacteriophages share 12 common proteins.
**Submitted**: 27 April 24; **Revised**: 30 September 24

**Table 38.** *Lindbergviridae*, 25 new taxa*

**Table:** 

Operation	Rank	New taxon name	Virus name	Exemplar
New taxon	Family	*Lindbergviridae*		
New taxon	Species	*Pbunavirus SG1*	Pseudomonas phage SG1	OQ594965
New taxon	Species	*Pbunavirus pv109*	Pseudomonas phage 109	OQ831730
New taxon	Species	*Pbunavirus FBPa14*	Pseudomonas phage vB_PaeM_FBPa14	ON375839
New taxon	Species	*Pbunavirus wadjak13*	Pseudomonas phage Kara-mokiny kep-wari Wadjak_13	OP310979
New taxon	Species	*Pbunavirus TH15*	Pseudomonas phage TH15	MW406974
New taxon	Species	*Pbunavirus PSA09*	Pseudomonas phage PSA09	MZ089730
New taxon	Species	*Pbunavirus ph0031*	Pseudomonas phage PhL_UNISO_PA-DSM_ph0031	MW526258
New taxon	Species	*Pbunavirus PSA25*	Pseudomonas phage PSA25	MZ089736
New taxon	Species	*Pbunavirus FBPa35*	Pseudomonas phage vB_PaeM_FBPa35	ON857938
New taxon	Species	*Pbunavirus victoria*	Pseudomonas phage Victoria	OR805296
New taxon	Species	*Myosmarvirus SMP*	Serratia phage SMP	OP490597
New taxon	Species	*Wifcevirus SP13*	Escherichia phage vB_EcoM_SP13	OP352608
New taxon	Species	*Wifcevirus AV128*	Escherichia phage AV128	OR352958
New taxon	Species	*Wifcevirus Ro157lw*	Escherichia phage vB_EcoM-Ro157lw	MH051335
New taxon	Species	*Wifcevirus EC150*	Escherichia phage EC150	ON210137
New taxon	Species	*Wifcevirus mansfield*	Escherichia phage Mansfield	MK903282
New taxon	Species	*Wifcevirus ECO71P1*	Escherichia phage ECO71P1	OP172789
New taxon	Species	*Carpasinavirus FoX6*	Xanthomonas phage FoX6	MT161386
New taxon	Genus	*Gladiolivirus*		
New taxon	Species	*Gladiolivirus maja*	Burkholderia phage Maja	MT708549
New taxon	Genus	*Irusalimvirus*		
New taxon	Species	*Irusalimvirus BCSR52*	Burkholderia phage BCSR52	MW460246
New taxon	Genus	*Plutovirus*		
New taxon	Species	*Plutovirus pluto*	Luteibacter phage vB_LflM-Pluto	ON529861

**Table 39.** *Lindbergviridae*, 7 move taxa*

**Table:** 

Operation	Rank	Taxon name	New parent taxon	Old parent taxa
Move taxon	Genus	*Kylevirus*	*Lindbergviridae*	*Caudoviricetes*
Move taxon	Genus	*Tabernariusvirus*	*Lindbergviridae*	*Caudoviricetes*
Move taxon	Genus	*Bcepfunavirus*	*Lindbergviridae*	*Caudoviricetes*
Move taxon	Genus	*Pbunavirus*	*Lindbergviridae*	*Caudoviricetes*
Move taxon	Genus	*Myosmarvirus*	*Lindbergviridae*	*Caudoviricetes*
Move taxon	Genus	*Wifcevirus*	*Lindbergviridae*	*Caudoviricetes*
Move taxon	Genus	*Carpasinavirus*	*Lindbergviridae*	*Caudoviricetes*

*Source/full text: https://ictv.global/ictv/proposals/2024.020B.Lindbergviridae_1nf_3ng_7mg_21ns.zip.

## 2024.021B.Malkevirus_1ng_5ns

**Title:** To create a new genus, *Malkevirus,* for *Streptococcus* prophages (class: *Caudoviricetes*)

**Authors:** Tolstoy I, Moraru C, Kropinski AM (Phage.Canada@gmail.com)

**Summary**

**Taxonomic rank(s) affected**
Realm *Duplodnaviria,* kingdom *Heunggongvirae,* phylum *Uroviricota,* class *Caudoviricetes.*
**Description of current taxonomy**
The viruses classified in this proposal do not have a current taxonomic assignment.
**Proposed taxonomic change(s)**
We propose a new genus, named in honour of Professor Dr. Horst Malke, comprised of temperate siphoviruses infecting *Streptococcus spp*.
**Justification**
The viruses fall into a genus based on current demarcation criteria.
**Submitted:** 07 May 24

**Table 40.** *Malkevirus*, 6 new taxa*

**Table:** 

Operation	Rank	New taxon name	Virus name	Exemplar
New taxon	Genus	*Malkevirus*		
New taxon	Species	*Malkevirus ARI02853*	Streptococcus phage phiARI0285-3	KT337347
New taxon	Species	*Malkevirus IPP67*	Streptococcus phage IPP67	KY065503
New taxon	Species	*Malkevirus IPP45*	Streptococcus phage IPP45	KY065485
New taxon	Species	*Malkevirus mv23782*	Streptococcus phage 23782	FR671408
New taxon	Species	*Malkevirus mv11865*	Streptococcus phage 11865	FR671409

*Source/full text: https://ictv.global/ictv/proposals/2024.021B.Malkevirus_1ng_5ns.zip.

## 2024.022B.Markadamsvirinae_1ng_1ns

**Title:** Create a new genus, *Kononvirus,* with a single species in the subfamily *Markadamsvirinae* (*Caudoviricetes: Demerecviridae*)

**Authors:** Wójcicki M (michal.wojcicki@hirszfeld.pl), Shymialevich D, Średnicka P, Gientka I, Błażejak S, Sokołowska B

**Summary**

**Taxonomic rank(s) affected**
Proposal to create a new genus (*Kononvirus*) with a single species (tailed phages from class *Caudoviricetes*, family *Demerecviridae* and subfamily *Markadamsvirinae*).
**Description of current taxonomy**
In 2022, significant changes to the taxonomy of bacterial viruses were introduced: the paraphyletic morphological families *Podoviridae*, *Siphoviridae* and *Myoviridae* as well as the order *Caudovirales* were abolished, which is replaced by the class *Caudoviricetes* to group all tailed bacterial and archaeal viruses with icosahedral capsids and a double-stranded DNA genome. Moreover, a binomial system of nomenclature for species was implemented. Currently, the family *Demerecviridae* includes three subfamilies (*Ermolyevavirinae, Markadamsvirinae* and *Mccorquodalevirinae*) and six separate genera (*Keyvirus, Novosibvirus, Pogseptimavirus, Priunavirus, Shenzhenvirus* and *Sugarlandvirus*) not classified at the subfamily level. The subfamily *Markadamsvirinae* currently includes two genera: *Epseptimavirus* and *Tequintavirus*.
**Proposed taxonomic change(s)**
We performed genomic analysis of newly isolated Enterobacter phage KKP_3711. Genome and protein analyses suggest that this bacteriophage belongs to the subfamily *Markadamsvirinae*. Still, the differences are too great to assign it to one of the two genera within this subfamily. Therefore, we propose to create a new genus, *Kononvirus,* including a single species, *Kononvirus KKP3711*.
**Justification**
Based on DNA and protein similarity, this is a cohesive genus.
**Submitted**: 16 June 24

**Table 41.** *Markadamsvirinae*, 2 new taxa*

**Table:** 

Operation	Rank	New taxon name	Virus name	Exemplar
New taxon	Genus	*Kononvirus*		
New taxon	Species	*Kononvirus KKP3711*	Enterobacter siphophage KKP_3711	PP579741

*Source/full text: https://ictv.global/ictv/proposals/2024.022B.Markadamsvirinae_1ng_1ns.zip.

## 2024.023B.Mcshanvirinae_1nsf_3ng_25ns

**Title:** To create a new subfamily, *Mcshanvirinae,* for *Streptococcus* prophages (class: *Caudoviricetes*)

**Authors:** Tolstoy I, Moraru C, Kropinski AM (Phage.Canada@gmail.com)

**Summary**

**Taxonomic rank(s) affected**
Realm *Duplodnaviria,* kingdom *Heunggongvirae,* phylum *Uroviricota,* class *Caudoviricetes.*
**Description of current taxonomy**
The viruses classified in this proposal do not have a current taxonomic assignment.
**Proposed taxonomic change(s)**
We propose a new subfamily, named in honour of Professor W. Michael McShan, of *Streptococcus* temperate siphoviruses containing three newly established genera: *Adrianbuildvirus, Medawarvirus* and *Phadecavirus*.
**Justification**
The proposed taxa conform to the demarcation criteria employed by the ICTV Bacterial Viruses Subcommittee.
**Submitted**: 06 May 2024

**Table 42.** *Mcshanvirinae*, 29 new taxa*

**Table:** 

Operation	Rank	New taxon name	Virus name	Exemplar
New taxon	Subfamily	*Mcshanvirinae*		
New taxon	Genus	*Adrianbuildvirus*		
New taxon	Species	*Adrianbuildvirus SpSL1*	Streptococcus phage SpSL1	KM882824
New taxon	Species	*Adrianbuildvirus IPP5*	Streptococcus phage IPP5	KY065449
New taxon	Species	*Adrianbuildvirus IPP44*	Streptococcus phage IPP44	KY065484
New taxon	Species	*Adrianbuildvirus IPP42*	Streptococcus phage IPP42	KY065482
New taxon	Species	*Adrianbuildvirus IPP51*	Streptococcus phage IPP51	KY065489
New taxon	Species	*Adrianbuildvirus ARI0923*	Streptococcus phage phiARI0923	KT337370
New taxon	Species	*Adrianbuildvirus IPP41*	Streptococcus phage IPP41	KY065481
New taxon	Species	*Adrianbuildvirus IPP43*	Streptococcus phage IPP43	KY065483
New taxon	Genus	*Medawarvirus*		
New taxon	Species	*Medawarvirus IPP12*	Streptococcus phage IPP12	KY065454
New taxon	Species	*Medawarvirus IPP22*	Streptococcus phage IPP22	KY065463
New taxon	Species	*Medawarvirus IPP18*	Streptococcus phage IPP18	KY065459
New taxon	Species	*Medawarvirus IPP57*	Streptococcus phage IPP57	KY065494
New taxon	Species	*Medawarvirus IPP20*	Streptococcus phage IPP20	KY065461
New taxon	Species	*Medawarvirus IPP21*	Streptococcus phage IPP21	KY065462
New taxon	Species	*Medawarvirus IPP30*	Streptococcus phage IPP30	KY065471
New taxon	Species	*Medawarvirus IPP11*	Streptococcus phage IPP11	KY065453
New taxon	Species	*Medawarvirus IPP29*	Streptococcus phage IPP29	KY065470
New taxon	Species	*Medawarvirus IPP19*	Streptococcus phage IPP19	KY065460
New taxon	Species	*Medawarvirus ARI01312*	Streptococcus phage phiARI0131-2	KT337342
New taxon	Species	*Medawarvirus IPP63*	Streptococcus phage IPP63	KY065499
New taxon	Species	*Medawarvirus IPP17*	Streptococcus phage IPP17	KY065458
New taxon	species	*Medawarvirus IPP28*	Streptococcus phage IPP28	KY065469
New taxon	Genus	*Phadecavirus*		
New taxon	Species	*Phadecavirus PH10*	Streptococcus phage PH10	FN391954
New taxon	Species	*Phadecavirus pv23TH*	Streptococcus phage 23TH	MT900487
New taxon	Species	*Phadecavirus olisA1*	Streptococcus phage OlisA1	OL774868

*Source/full text: https://ictv.global/ictv/proposals/2024.023B.Mcshanvirinae_1nsf_3ng_25ns.zip.

## 2024.024B.Mtkvariviridae_1nf_1msf_10ns

**Title:** Create a new family, *Mtkvariviridae,* for PhiEco32-like phages (class: *Caudoviricetes*)

**Authors:** Moraru C, Tolstoy I, Kropinski AM (Phage.Canada@gmail.com)

**Summary**

**Taxonomic rank(s) affected**
Realm *Duplodnaviria,* kingdom *Heunggongvirae,* phylum *Uroviricota,* class *Caudoviricetes.*
**Description of current taxonomy**
The genera *Kuravirus, Nieuwekanaalvirus* and *Suseptimavirus* are currently classified within the subfamily *Gordonclarkvirinae,* class *Caudoviricetes.*
**Proposed taxonomic change(s)**
Create eight new species in the genus *Kuravirus.*
Create two new species in the genus *Suseptimavirus.*
Create a new family, *Mtkvariviridae.*
**Justification**
Species classified to the genera *Kuravirus, Nieuwekanaalvirus* and *Suseptimavirus* within the subfamily *Gordonclarkvirinae* share 12 core proteins and form a deep branching clade in ViPTree tBLASTx distance analysis.
**Submitted:** 06 May 24; **Revised:** 30 September 24

**Table 43.** *Mtkvariviridae*, 11 new taxa*

**Table:** 

Operation	Rank	New taxon name	Virus name	Exemplar
New taxon	Family	*Mtkvariviridae*		
New taxon	Species	*Kuravirus myPSH1131*	Escherichia phage myPSH1131	MG983840
New taxon	Species	*Kuravirus myPSH2311*	Escherichia phage myPSH2311	MG976803
New taxon	Species	*Kuravirus XT18*	Escherichia phage vB-EcoP-XT18	OR757434
New taxon	Species	*Kuravirus LAMP*	Escherichia phage LAMP	MG673519
New taxon	Species	*Kuravirus SDYTW1F1223*	Escherichia phage SDYTW1-F1-2-2_3	OR296290
New taxon	Species	*Kuravirus pECN12032Af1*	Escherichia phage pEC-N1203-2Af.1	OQ540978
New taxon	Species	*Kuravirus YF01*	Escherichia phage vB_EcoP_YF01	OQ025076
New taxon	Species	*Kuravirus SR02*	Escherichia phage SR02	OQ870566
New taxon	Species	*Suseptimavirus PAS59*	Escherichia phage vB_EcoP_PAS59	OQ921332
New taxon	Species	*Suseptimavirus sv4E8*	Escherichia phage 4E8	OQ689734

**Table 44.** *Mtkvariviridae*, 1 move taxon*

**Table:** 

Operation	Rank	Taxon name	New parent taxon	Old parent taxon
Move taxon	Subfamily	*Gordonclarkvirinae*	*Mtkvariviridae*	*Caudoviricetes*

*Source/full text: https://ictv.global/ictv/proposals/2024.024B.Mtkvariviridae_1nf_1msf_10ns.zip.

## 2024.025B.Obscuriviridae_1nf_2ng_3ns

**Title:** Create a new family, *Obscuriviridae*

**Authors:** Bartlau N (nina.bartlau@univie.ac.at), Moraru C, Wichels A, Holmfeldt K, Amann RI

**Summary**

**Taxonomic rank(s) affected**
New, unassigned family.
**Description of current taxonomy**
The viruses described in this proposal do not have a current taxonomic assignment.
**Proposed taxonomic change(s)**
A new family, *Obscuriviridae,* is proposed, including two new genera, *Omtjevirus* and *Cebaduodecimvirus*.
**Justification**
The *Obscuriviridae* family was delineated with two genera. The genus *Omtjevirus* has *Omtjevirus Omtje* as a species and the genus *Cebaduodecimvirus* has *Cebaduodecimvirus phi12duo* and *Cebaduodecimvirus phi12auna* as species. The proposed taxonomic assignments are based on six different methods including nucleotide-, protein-, amino acid- and core protein-based analysis.
**Submitted**: 21 June 24; **Revised**: 30 September 24

**Table 45.** *Obscuriviridae*, 6 new taxa*

**Table:** 

Operation	Rank	New taxon name	Virus name	Exemplar
New taxon	Family	*Obscuriviridae*		
New taxon	Genus	*Omtjevirus*		
New taxon	Species	*Omtjevirus Omtje*	Cellulophaga phage Omtje_1	MT732445
New taxon	Genus	*Cebaduodecimvirus*		
New taxon	Species	*Cebaduodecimvirus phi12duo*	Cellulophaga phage phi12 : 2	KC821606
New taxon	Species	*Cebaduodecimvirus phi12auna*	Cellulophaga phage phi12a:1	KC821623

*Source/full text: https://ictv.global/ictv/proposals/2024.025B.Obscuriviridae_1nf_2ng_3ns.zip.

## 2024.026B.Pantevenvirales_1no_3mf

**Title:** Create one new order *Pantevenvirales* (*Duplodnaviria*)

**Authors:** Adriaenssens EM (Evelien.adriaenssens@quadram.ac.uk), Cook R, Millard AD, Turner D

**Summary**

**Taxonomic rank(s) affected**
Realm: *Duplodnaviria*; kingdom: *Heunggongvirae*; phylum: *Uroviricota*; class: *Caudoviricetes.*
**Description of current taxonomy**
The families *Straboviridae, Kyanoviridae* and *Ackermannviridae* are unassigned at the order rank within the class *Caudoviricetes*.
**Proposed taxonomic change(s)**
The order *Pantevenvirales* is created for these families.
**Justification**
The families *Straboviridae, Kyanoviridae* and *Ackermannviridae* form a monophyletic cluster in proteome-based analyses. Members of these families share a number of core orthologous genes.
**Submitted**: 21 June 24

**Table 46.** *Pantevenvirales*, 3 move taxa*

**Table:** 

Operation	Rank	Taxon name	New parent taxon	Old parent taxon
Move taxon	Family	*Straboviridae*	*Pantevenvirales*	*Caudoviricetes*
Move taxon	Family	*Kyanoviridae*	*Pantevenvirales*	*Caudoviricetes*
Move taxon	Family	*Ackermannviridae*	*Pantevenvirales*	*Caudoviricetes*

**Table 47.** *Pantevenvirales*, 1 new taxon*

**Table:** 

Operation	Rank	New taxon name
New taxon	Order	*Pantevenvirales*

*Source/full text: https://ictv.global/ictv/proposals/2024.026B.Pantevenvirales_1no_3mf.zip.

## 2024.028B.Philemonvirus_1ns

**Title:** To create one new species in the genus *Bifilivirus,* family *Paulinoviridae*

**Authors:** Deptula P (deptula@food.ku.dk), Sha Y, Potipimpanon S, Vogensen FK, Nielsen DS, Knezevic P

**Summary**

**Taxonomic rank(s) affected**
This is a proposal for creating a new species within the genus *Bifilivirus*.
**Description of current taxonomy**
The genus *Bifilivirus* currently includes one species.
**Proposed taxonomic change(s)**
Addition of a new species *Bifilivirus philemonii.*
**Justification**
Filamentous phage Philemon was isolated from raw milk Emmental cheese on a dairy-associated strain of *Propionibacterium freudenreichii* PB4. The obtained TEM confirmed filamentous morphology. Philemon forms plaques on three *P. freudenreichii* strains in our collection, including the strain TL18, which was used for characterization of phage B5 (though no plaquing was reported for phage B5) [12]. The phage genome was sequenced with the Illumina platform after the formation of the complementary strand with the MDA technique. The genome is 5802 nucleotides, with 63 G+C %mol. Comparison with the only other representative of genus *Bifilivirus,* Propionibacterium virus B5 (AF428260), revealed that the genome of Philemon is 92.8% identical to the genome of the Propionibacterium virus B5, thus fulfilling the criteria for the creation of a separate species. The similarity of morphogenesis protein and CoaB confirms that Philemon belongs to the genus *Bifilivirus*. We propose the name of the species – *Bifivilus philemonii*.
**Submitted:** 23 April 24

**Table 48.** *Philemonvirus*, 1 new taxon*

**Table:** 

Operation	Rank	New taxon name	Virus name	Exemplar
New taxon	Species	*Bifilivirus philemonii*	Propionibacterium phage Philemon	PP693361

*Source/full text: https://ictv.global/ictv/proposals/2024.028B.Philemonvirus_1ns.zip.

## 2024.029B.Rhodococcus_siphoviruses_7ng_7ns

**Title:** Create seven new genera for *Rhodococcus* siphoviruses (class: *Caudoviricetes*)

**Authors:** Kurtböke I, Moraru C, Tolstoy I, Kropinski AM (Phage.Canada@gmail.com)

**Summary**

**Taxonomic rank(s) affected**
Realm *Duplodnaviria,* kingdom *Heunggongvirae,* phylum *Uroviricota,* class *Caudoviricetes.*
**Description of current taxonomy**
The viruses classified in this proposal do not have a current taxonomic assignment.
**Proposed taxonomic change(s)**
We propose the creation of seven new taxa for unique *Rhodococcus* siphoviruses: *Edwardsroadvirus, Wodongavirus, Reqipinevirus, Melbournevirus, Trogglehumpervirus, Mboduovirus, Reynauldvirus.*
**Justification**
The taxa proposed conform to demarcation criteria specified by the ICTV Bacterial Viruses Subcommittee.
**Submitted**: 09 May 2024

**Table 49.** *Rhodococcus* siphoviruses, 14 new taxa*

**Table:** 

Operation	Rank	New taxon name	Virus name	Exemplar
New taxon	Genus	*Edwardsroadvirus*		
New taxon	Species	*Edwardsroadvirus RRH1*	Rhodococcus phage RRH1	JN116822
New taxon	Genus	*Wodongavirus*		
New taxon	Species	*Wodongavirus REQ3*	Rhodococcus phage REQ3	JN116824
New taxon	Genus	*Reqipinevirus*		
New taxon	Species	*Reqipinevirus reqipine5*	Rhodococcus phage ReqiPine5	GU580943
New taxon	Genus	*Melbournevirus*		
New taxon	Species	*Melbournevirus REQ2*	Rhodococcus phage REQ2	JN116823
New taxon	Genus	*Trogglehumpervirus*		
New taxon	Species	*Trogglehumpervirus trogglehumper*	Rhodococcus phage Trogglehumper	OQ709222
New taxon	Genus	*Mboduovirus*		
New taxon	Species	*Mboduovirus mbo2*	Rhodococcus phage Mbo2	ON191531
New taxon	Genus	*Reynauldvirus*		
New taxon	Species	*Reynauldvirus reynauld*	Rhodococcus phage Reynauld	OR159659

*Source/full text: https://ictv.global/ictv/proposals/2024.029B.Rhodococcus_siphoviruses_7ng_7ns.zip.

## 2024.030B.Trautnerviridae_1nf_1nsf_3ng_6ns

**Title:** Create a new family, *Trautnerviridae,* subfamily *Polsinellivirinae* and two genera (*Rivavirus,* and *Splendidredvirus,* class *Caudoviricetes*)

**Authors:** Cook R, Tavares P, Lurz R, Barylski J, Moraru C, Tolstoy I, Kropinski AM (Phage.Canada@gmail.com)

**Summary**

**Taxonomic rank(s) affected**
Realm *Duplodnaviria,* kingdom *Heunggongvirae,* phylum *Uroviricota,* class *Caudoviricetes.*
**Description of current taxonomy**
The viruses classified in this proposal do not have a current taxonomic assignment.
**Proposed taxonomic change(s)**
To create a new genus, *Rivavirus*, with three species.
To create a new genus, *Splendidredvirus*, with two species.
To create a new subfamily, *Polsinellivirinae*, with these two genera (*Rivavirus* and *Splendidredvirus*).
To create a new single-species genus, *Prospektnaukivirus.*
To create a new family, *Trautnerviridae,* for these taxa.
**Justification**
Bacillus phage SPP1 was isolated in 1966; sequenced in 1997 (corrected in 2018); and has been the subject of numerous morphological and physiological studies; yet has remained unclassified. In this proposal, it has been assigned to a new genus, *Rivavirus,* together with Bacillus phage vB_BspS_SplendidRed (assigned to the new genus *Splendidredvirus),* to form a new subfamily, *Polsinellivirinae*. The members of this taxon are siphoviruses which have genomes of 42.8–46.3 kb (43.7–44.6 mol% G+C) and encode 74–77 proteins and no tRNAs. As a result of detailed genomic, proteomic and phylogenetic analyses using VIRIDIC, ViPTree and VirClust, we further propose to create a new family for these genera named *Trautnerviridae* named in honour of Thomas A. Trautner.
**Submitted**: 25 May 24; **Revised**: 30 September 24

**Table 50.** *Trautnerviridae*, 11 new taxa*

**Table:** 

Operation	Rank	New taxon name	Virus name	Exemplar
New taxon	Family	*Trautnerviridae*		
New taxon	Subfamily	*Polsinellivirinae*		
New taxon	Genus	*Rivavirus*		
New taxon	Species	*Rivavirus SPP1*	Bacillus phage SPP1	X97918
New taxon	Species	*Rivavirus rv000TH010*	Bacillus phage 000TH010	MN176219
New taxon	Species	*Rivavirus rv049ML001*	Bacillus phage 049 ml001	MN176227
New taxon	Genus	*Splendidredvirus*		
New taxon	Species	*Splendidredvirus splendidred*	Bacillus phage vB_BspS_SplendidRed	MN013088
New taxon	Species	*Splendidredvirus ray17*	Bacillus phage Ray17	MH752385
New taxon	Genus	*Prospektnaukivirus*		
New taxon	Species	*Prospektnaukivirus sam112*	Bacillus phage vB_BcM_Sam112	MN604230

*Source/full text: https://ictv.global/ictv/proposals/2024.030B.Trautnerviridae_1nf_1nsf_3ng_6ns.zip.

## 2024.031B.Sarkviridae_1nf_1msf_2mg

**Title:** Create a new family, *Sarkviridae* for the Jersey-like siphophages (class: *Caudoviricetes*)

**Authors:** Moraru C, Tolstoy I, Kropinski AM (Phage.Canada@gmail.com)

**Summary**

**Taxonomic rank(s) affected**
Realm *Duplodnaviria,* kingdom *Heunggongvirae,* phylum *Uroviricota,* class *Caudoviricetes.*
**Description of current taxonomy**
The genera *Jerseyvirus, Cornellvirus and Kagunavirus* in the subfamily *Guernseyvirinae* and among the *Serratia* phages the genera *Seretavirus* and *Otakuvirus.*
**Proposed taxonomic change(s)**
To create a new family *Sarkviridae.*
**Justification**
Genomic, proteomic and phylogenetic data indicate that this group of phages is a family. In addition, Taxonomy Proposal 2023.068B.Caudoviricetes_Serratia_3ng suggested a higher level relationship between the taxa *Otakuvirus* and *Guernseyvirinae*.
**Submitted**: 04 June 24; **Revised**: 30 September 24

**Table 51.** *Sarkviridae*, 3 move taxa*

**Table:** 

Operation	Rank	Taxon name	New parent taxon	Old parent taxon
Move taxon	Subfamily	*Guernseyvirinae*	*Sarkviridae*	*Caudoviricetes*
Move taxon	Genus	*Seretavirus*	*Sarkviridae*	*Caudoviricetes*
Move taxon	Genus	*Otakuvirus*	*Sarkviridae*	*Caudoviricetes*

**Table 52.** *Sarkviridae*, 1 new taxon*

**Table:** 

Operation	Rank	New taxon name
New taxon	Family	*Sarkviridae*

*Source/full text: https://ictv.global/ictv/proposals/2024.031B.Sarkviridae_1nf_1msf_2mg.zip.

## 2024.032B.Sepahanvirus_1ng_2ns

**Title:** Create a new genus, *Sepahanvirus,* containing two species (*Caudoviricetes*)

**Authors:** Ganjoor MS, Bouzari M (bouzari@sci.ui.ac.ir), Soleimani-Delfan A

**Summary**

**Taxonomic rank(s) affected**
Genus.
**Description of current taxonomy**
These phages are currently unclassified.
**Proposed taxonomic change(s)**
To create a new genus, *Sepahanvirus,* within the class *Caudoviricetes* comprising two species, for Yersinia phage vB_Yru_GN1 and Yersinia phage YerA41.
**Justification**
Yersinia phage vB_Yru_GN1 and Yersinia phage YerA41 exhibit nucleotide sequence similarity that falls within the demarcation threshold for the creation of a new genus.
**Submitted**: 09 December 23

**Table 53.** *Sepahanvirus*, 3 new taxa*

**Table:** 

Operation	Rank	New taxon name	Virus name	Exemplar
New taxon	Genus	*Sepahanvirus*		
New taxon	Species	*Sepahanvirus GN1*	Yersinia phage vB_Yru_GN1	LC779065
New taxon	Species	*Sepahanvirus yerA41*	Yersinia phage YerA41	MW570730

*Source/full text: https://ictv.global/ictv/proposals/2024.032B.Sepahanvirus_1ng_2ns.zip.

## 2024.033B.Mazoviaviridae_1nf_1ng_1ns

**Title:** Create a new family, *Mazoviaviridae,* and a new genus, *Dabrowskivirus,* with a single species (class *Caudoviricetes*)

**Authors:** Shymialevich D, Wójcicki M (michal.wojcicki@hirszfeld.pl), Sokołowska B

**Summary**

**Taxonomic rank(s) affected**
Proposal to create a new family, *Mazoviaviridae,* and a new genus, *Dabrowskivirus,* with a single species (class *Caudoviricetes*).
**Description of current taxonomy**
In 2022, significant changes to the taxonomy of bacterial viruses were introduced: the paraphyletic morphological families *Podoviridae*, *Siphoviridae* and *Myoviridae* as well as the order *Caudovirales* were abolished, which is replaced by the class *Caudoviricetes* to group all tailed bacterial and archaeal viruses with icosahedral capsids and a double-stranded DNA genome. Moreover, a binomial system of nomenclature for species was established. Based on the morphology and the comparative analysis of its predicted proteins, Alicyclobacillus myophage vB_Aac_IAFB_3916 was assigned to viruses with complex structures (class *Caudoviricetes*).
**Proposed taxonomic change(s)**
Analyses of the phylogenetic relationship of Alicyclobacillus myophage vB_Aac_IAFB_3916 were unable to provide an unambiguous assignment to a specific family and genus. The weak similarity with other phage genomes deposited in the databases suggests that the isolated bacteriophage may be a representative of a new genus and new family of tailed bacteriophages.
**Justification**
The genome of newly isolated Alicyclobacillus myophage vB_Aac_IAFB_3916 possesses no DNA homologues. At the protein level, this virus is unique. Therefore, we propose the creation of a new species (*Dabrowskivirus KKP3916*)*,* genus (*Dabrowskivirus*) and family (*Mazoviaviridae*) for viruses of this type.
**Submitted**: 09 June 24; **Revised:** 09 October 24

**Table 54.** *Mazoviaviridae*, 3 new taxa*

**Table:** 

Operation	Rank	New taxon name	Virus name	Exemplar
New taxon	Family	*Mazoviaviridae*		
New taxon	Genus	*Dabrowskivirus*		
New taxon	Species	*Dabrowskivirus KKP3916*	Alicyclobacillus myophage vB_Aac_IAFB_3916	OQ846916

*Source/full text: https://ictv.global/ictv/proposals/2024.033B.Mazoviaviridae_1nf_1ng_1ns.zip.

## 2024.034B.Stackebrandtviridae_1nf_2nsf_8mg_8ns

**Title:** Create a new family, *Stackebrandtviridae,* for a group of *Gordonia* phages (class: *Caudoviricetes*)

**Authors:** Kurtböke I, Moraru C, Tolstoy I, Kropinski AM (Phage.Canada@gmail.com)

**Summary**

**Taxonomic rank(s) affected**
Realm *Duplodnaviria,* kingdom *Heunggongvirae,* phylum *Uroviricota,* class *Caudoviricetes.*
**Description of current taxonomy**
At present, the following taxa exist as floating genera in the class *Caudoviricetes; Wizardvirus, Clownvirus, Vividuovirus, Dexdertvirus, Zitchvirus*, *Kroosvirus* and *Leonardvirus.*
**Proposed taxonomic change(s)**
To create one new species in the genus *Wizardvirus.*
To create a new subfamily, *Frickvirinae* with two genera (*Clownvirus* and *Wizardvirus*).
To add one new species to the genus *Vividuovirus.*
To add one new species to the genus *Dexdertvirus.*
To add four new species to the genus *Zitchvirus.*
To add one new species to the genus *Leonardvirus.*
To create a new subfamily, *Schenleyvirinae,* for the above four genera and *Kroosvirus*.
To create a new family, *Stackebrandtviridae,* for the above-mentioned taxa.
**Justification**
Members of the Actinobacteriophage Database Cluster DC (https://phagesdb.org/clusters/DC/) are temperate *Gordonia* phages for which we have created two genera. The related lytic viruses of Cluster DE (https://phagesdb.org/clusters/DE/) have previously been placed in five genera.
**Submitted:** 15 June 24; **Revised:** 30 September 24

**Table 55.** *Stackebrandtviridae*, 11 new taxa*

**Table:** 

Operation	Rank	New taxon name	Virus name	Exemplar
New taxon	Family	*Stackebrandtviridae*		
New taxon	Subfamily	*Frickvirinae*		
New taxon	Species	*Wizardvirus halo3*	Gordonia phage Halo3	OR521081
New taxon	Subfamily	*Schenleyvirinae*		
New taxon	Species	*Vividuovirus sitar*	Gordonia phage Sitar	MH153809
New taxon	Species	*Dexdertvirus kwekel*	Gordonia phage Kwekel	OR521074
New taxon	Species	*Zitchvirus tardus*	Gordonia phage Tardus	ON392159
New taxon	Species	*Zitchvirus viaconlectus*	Gordonia phage ViaConlectus	OP068342
New taxon	Species	*Zitchvirus sampson*	Gordonia phage Sampson	ON456337
New taxon	Species	*Zitchvirus apunk*	Gordonia phage APunk	ON755186
New taxon	Species	*Leonardvirus phauci*	Gordonia phage Phauci	ON456349

**Table 56.** *Stackebrandtviridae*, 8 move taxa*

**Table:** 

Operation	Rank	Taxon name	New parent taxon	Old parent taxon
Move taxon	Genus	*Clownvirus*	*Frickvirinae*	*Caudoviricetes*
Move taxon	Genus	*Wizardvirus*	*Stackebrandtviridae*	*Caudoviricetes*
Move taxon	Genus	*Kroosvirus*	*Schenleyvirinae*	*Caudoviricetes*
Move taxon	Genus	*Vividuovirus*	*Schenleyvirinae*	*Caudoviricetes*
Move taxon	Genus	*Dexdertvirus*	*Schenleyvirinae*	*Caudoviricetes*
Move taxon	Genus	*Zitchvirus*	*Schenleyvirinae*	*Caudoviricetes*
Move taxon	Genus	*Leonardvirus*	*Schenleyvirinae*	*Caudoviricetes*
Move taxon	Genus	*Lilbeanievirus*	*Stackebrandtviridae*	*Caudoviricetes*

*Source/full text: https://ictv.global/ictv/proposals/2024.034B.Stackebrandtviridae_1nf_2nsf_8mg_8ns.zip.

## 2024.036B.Caudoviricetes_Faserviricetes_Name_Corrections

**Title:** Corrections to species names in the classes *Caudoviricetes* and *Faserviricetes*

**Authors:** Turner D (dann2.turner@uwe.ac.uk)

**Summary**

**Taxonomic rank(s) affected**
Genera and species in realm *Duplodnaviria,* kingdom *Heunggongvirae,* phylum *Uroviricota,* class *Caudoviricetes.*
**Description** **of current taxonomy**
All of these species are currently classified within the latest release of the ICTV taxonomy.
**Proposed taxonomic change(s)**
Rename species to conform to the binomial species epithet.
Correct spelling errors.
Create genus to contain a floating species in the subfamily *Tevenvirinae.*
Change to genus name and constituent species in the genus *Roskildevirus* as ‘Roskilde virus’ refers to Norovirus in Danish.
Correction of misspelt genus names in binomial species epithets.
Move two species that were incorrectly classified within the genus *Tequatrovirus*.
Abolish species *Campylobacter virus IBB35.*
**Justification**
To ensure that the naming of viruses is consistent with the guidelines for binomial species names, to remove any names that could cause confusion in native languages and to remove any genome records that do not represent coding complete sequences.
**Submitted**: 19 June 24; **Revised**: 30 September 24

**Table 57.** *Caudoviricetes*, 96 rename taxa*. Table too large, see supplementary information sheet supp_info_tab_57

**Table 58.** *Caudoviricetes*, 3 move; rename taxa*

**Table:** 

Operation	Rank	New taxon name	Old parent taxon	New parent taxon	Old taxon name
Move; rename taxon	Species	*Centumtrigintavirus cv133*	*Tevenvirinae*	*Centumtrigintavirus*	*Acinetobacter virus 133*
Move; rename taxon	Species	*Mosigvirus jaykay*	*Tequatrovirus*	*Mosigvirus*	*Tequatrovirus jaykay*
Move; rename taxon	Species	*Mosigvirus efftwo*	*Tequatrovirus*	*Mosigvirus*	*Tequatrovirus efftwo*

**Table 59.** *Caudoviricetes*, 1 abolish taxon*

**Table:** 

Operation	Rank	Abolished taxon name
Abolish taxon	Species	*Campylobacter virus IBB35*

**Table 60.** *Caudoviricetes*, 1 new taxon*

**Table:** 

Operation	Rank	New taxon name
New taxon	Genus	*Centumtrigintavirus*

*Source/full text: https://ictv.global/ictv/proposals/2024.036B.Caudoviricetes_Faserviricetes_Name_Corrections.zip.

## 2024.037B.Vandenendeviridae_1nf_2msf_8ng_1mg_11ns

**Title:** Create a new family, *Vandenendeviridae,* for a group of lytic *Pseudomonas* phages (class: *Caudoviricetes*)

**Authors:** Moraru C, Tolstoy I, Kropinski AM (Phage.Canada@gmail.com)

**Summary**

**Taxonomic rank(s) affected**
Realm *Duplodnaviria,* kingdom *Heunggongvirae,* phylum *Uroviricota,* class *Caudoviricetes.*
**Description of current taxonomy**
Five genera are currently classified; *Baldwinvirus, Nankokuvirus, Otagovirus*, *Flaumdravirus* and *Pakpunavirus*.
**Proposed taxonomic change(s)**
To create seven new single-species genera: *Weillhallvirus, Omahavirus, Torinovirus, Yunamivirus, Ventosusvirus*, *Uavernvirus* and *Chemalvirus.*
To create a new genus, *Tartuvirus,* with four species.
To create two new species in the genus *Kremarvirus.*
To create a new family, *Vandenendeviridae,* for these genera and *Baldwinvirus, Kremarvirus, Nankokuvirus, Otagovirus, Flaumdravirus*, *Pakpunavirus* and *Shenlongvirus*.
**Justification**
Using VIRIDIC, ViPTree, VIRCLUST and vConTACT v.3.0, we have established that this is a cohesive group of lytic Pseudomonas myoviruses which share ≥12.2% DNA sequence similarity and 15 core proteins. The new family is named in honour of Marius van de Ende of South Africa.
**Submitted**: 25 May 24; **Revised**: 30 September 24

**Table 61.** *Vandenendeviridae*, 20 new taxa*

**Table:** 

Operation	Rank	New taxon name	Virus name	Exemplar
New taxon	Family	*Vandenendeviridae*		
New taxon	Genus	*Weillhallvirus*		
New taxon	Species	*Weillhallvirus wv16Q*	Pseudomonas phage 16Q	OR001909
New taxon	Genus	*Omahavirus*		
New taxon	Species	*Omahavirus UNOG1W1*	Pseudomonas phage UNO-G1W1	PP551948
New taxon	Genus	*Torinovirus*		
New taxon	Species	*Torinovirus K7A1*	Pseudomonas phage phiK7A1	MT740307
New taxon	Genus	*Yunamivirus*		
New taxon	Species	*Yunamivirus Y1MI*	Pseudomonas phage vB_PF_Y1-MI	OR500437
New taxon	Genus	*Ventosusvirus*		
New taxon	Species	*Ventosusvirus ventosus*	Pseudomonas phage ventosus	MG018930
New taxon	Genus	*Uavernvirus*		
New taxon	Species	*Uavernvirus uavern*	Pseudomonas phage UAVern	MZ605293
New taxon	Genus	*Tartuvirus*		
New taxon	Species	*Tartuvirus amme3*	Pseudomonas phage vB_PpuM-Amme-3	PP496413
New taxon	Species	*Tartuvirus nopa*	Pseudomonas phage vB_PpuM-NoPa	PP496415
New taxon	Species	*Tartuvirus kopa4*	Pseudomonas phage vB_PpuM-KoPa-4	PP496414
New taxon	Species	*Tartuvirus roomu2*	Pseudomonas phage vB_PpuM-Roomu-2	PP496417
New taxon	Genus	*Chemalvirus*		
New taxon	Species	*Chemalvirus PseuGes254*	Pseudomonas phage PseuGes_254	OR575930

**Table 62.** *Vandenendeviridae*, 3 move taxa*

**Table:** 

Operation	Rank	Taxon name	New parent taxon	Old parent taxon
Move taxon	Subfamily	*Skurskavirinae*	*Vandenendeviridae*	*Caudoviricetes*
Move taxon	Subfamily	*Gorskivirinae*	*Vandenendeviridae*	*Caudoviricetes*
Move taxon	Genus	*Nankokuvirus*	*Vandenendeviridae*	*Caudoviricetes*

*Source/full text: https://ictv.global/ictv/proposals/2024.037B.Vandenendeviridae_1nf_2msf_8ng_1mg_11ns.zip.

## 2024.038B.Vinavirales_3nf_1mf_7ng_5ns_3ms

**Title:** Create three new families *Mestraviridae, Asemoviridae* and *Parnassusviridae,* and move the family *Autolykiviridae* into the order *Vinavirales (Tectiliviricetes, Preplasmiviricota, Bamfordvirae, Varidnaviria*)

**Authors:** Bardy P, Fogg PCM, Kalatzis PG, Middelboe M, Oksanen HM (hanna.oksanen@helsinki.fi)

**Summary**

**Taxonomic rank(s) affected**
The taxonomic ranks affected are the genus *Corticovirus* in the family *Corticoviridae,* the order *Vinavirales* and the family *Autolykiviridae*. In addition, some species are affected.
**Description of current taxonomy**
Currently, the order *Vinavirales* includes one family *Corticoviridae* with one genus *Corticovirus* (two species). The family *Autolykiviridae* belongs to the class *Tectiliviricetes* but is not assigned to any order. The family *Autolykiviridae* includes two genera *Livvievirus* (two species) and *Paulavirus* (three species). The realm *Varidnaviria* to which these taxa are classified has been reorganized in proposal 2024.010D.
**Proposed taxonomic change(s)**
The genus *Corticovirus* (family *Corticoviridae*) is renamed as the genus *Merivirus* and its two species are renamed accordingly. The *Vinavirales* order is rearranged so that the family *Autolykiviridae* is placed in the order along with the family *Corticoviridae*. Two new genera, *Oliviavirus* and *Ameliavirus,* are created in the family *Autolykiviridae*, each with a single species, *Oliviavirus viph1020o* and *Ameliavirus viph1008o,* respectively. The genus of the virus species *Paulavirus viph1044o* is moved from *Livvievirus* to *Paulavirus* (family *Autolykiviridae*). In addition, three new families *Mestraviridae, Asemoviridae* and *Parnassusviridae* are created in the order *Vinavirales*. Two new genera, *Anticleavirus* and *Polymedevirus,* are created in the family *Mestraviridae*. One new species is created in each of two genera: *Anticleavirus jorvik* and *Polymedevirus YY*. Two new genera, *Elsinorevirus* and *Rumoivirus,* are created in the family *Asemoviridae*. One new species is created in each of two genera: *Elsinorevirus NO16* and *Rumoivirus VruC*. One new species *Corycianvirus MfV* is created in a new genus *Corycianvirus* in the family *Parnassusviridae*.
**Justification**
The inclusion/creation of four families in the order *Vinavirales,* together with the family *Corticoviridae,* is based on the nine signature genes shared by their members, which corresponds to approximately 50% of their genome. This demonstrates their common evolutionary origin. Classification of *Paulavirus viph1044o* into the genus *Livvievirus* instead of *Paulavirus* was a mistake. To make the names of the family *Corticoviridae* and the genus *Corticovirus* based on a different word stem, the genus *Corticovirus* is renamed *Merivirus*.
**Submitted**: 20 June 24; **Revised**: 30 September 24

**Table 63.** *Vinavirales*, 15 new taxa*

**Table:** 

Operation	Rank	New taxon name	Virus name	Exemplar
New taxon	Genus	*Oliviavirus*		
New taxon	Genus	*Ameliavirus*		
New taxon	Family	*Mestraviridae*		
New taxon	Genus	*Anticleavirus*		
New taxon	Species	*Anticleavirus jorvik*	Rhodobacter phage Jorvik	OP588643
New taxon	Genus	*Polymedevirus*		
New taxon	Species	*Polymedevirus YY*	Marinomonas phage YY	MH105080
New taxon	Family	*Asemoviridae*		
New taxon	Genus	*Elsinorevirus*		
New taxon	Species	*Elsinorevirus NO16*	Vibrio phage fNo16	MH730557
New taxon	Genus	*Rumoivirus*		
New taxon	Species	*Rumoivirus VruC*	Vibrio phage vB_VruC_PG21	OM867525
New taxon	Family	*Parnassusviridae*		
New taxon	Genus	*Corycianvirus*		
New taxon	Species	*Corycianvirus MfV*	Marinomonas phage MfV	MW618650

**Table 64.** *Vinavirales*, 2 move taxa and 2 move rename taxa*

**Table:** 

Operation	Rank	Taxon name	New parent taxon	Old parent taxon	Old taxon name
Move taxon	Family	*Autolykiviridae*	*Vinavirales*	*Tectiliviricetes*	
Move taxon	Species	*Paulavirus viph1044o*	*Paulavirus*	*Livvievirus*	
Move; rename taxon	Species	*Oliviavirus viph1020o*	*Oliviavirus*	*Paulavirus*	*Paulavirus viph1020o*
Move; rename taxon	Species	*Ameliavirus viph1008o*	*Ameliavirus*	*Paulavirus*	*Paulavirus viph1008o*

**Table 65.** *Vinavirales*, 3 rename taxa*

**Table:** 

Operation	Rank	New taxon name	Previous taxon name
Rename taxon	Genus	*Merivirus*	*Corticovirus*
Rename taxon	Species	*Merivirus Cr39582*	*Corticovirus Cr39582*
Rename taxon	Species	*Merivirus PM2*	*Corticovirus PM2*

*Source/full text: https://ictv.global/ictv/proposals/2024.038B.Vinavirales_3nf_1mf_7ng_5ns_3ms.zip.

## 2024.039B.Artimaviricota_np

**Title:** Create new phylum, *Artimaviricota* in the kingdom *Orthornavirae* (realm *Riboviria*) for classification of a hyperthermophilic RNA virus

**Authors:** Syun-ichi Urayama (urayama.shunichi.gn@u.tsukuba.ac.jp), Akihito Fukudome, Eugene V. Koonin, Takuro Nunoura, Mart Krupovic (mart.krupovic@pasteur.fr)

**Summary**

**Taxonomic rank(s) affected**
*Riboviria, Orthornavirae.*
**Description of current taxonomy**
Realm *Riboviria* includes two kingdoms, *Orthornavirae* and *Pararnavirae,* which include highly diverse viruses that encode RNA-directed RNA polymerases (RdRP) and reverse transcriptases (RT), respectively. Kingdom *Orthornavirae* includes six phyla which were established based on phylogenetic analysis of the RdRP and comparative analysis of the viral genomes and proteins.
**Proposed taxonomic change(s)**
We propose to create a new phylum in the kingdom *Orthornavirae* for classification of a group of RNA viruses discovered in hot springs that are characterized by unusual RdRPs.
**Justification**
The RdRPs of hot spring RNA virus 1 and its relatives seem to deviate from the RdRP consensus farther than any of the other recently discovered putative phyla, with none of which they appear to be affiliated, and possess unusual structural features that appear to link them to viral RTs.
**Submitted**: 21 June 2024

**Table 66.** *Artimaviricota*, 6 new taxa*

**Table:** 

Operation	Rank	New taxon name	Virus name	Exemplar
New taxon	Phylum	*Artimaviricota*		
New taxon	Class	*Furtirnaviricetes*		
New taxon	Order	*Divaquavirales*		
New taxon	Family	*Hakuzoviridae*		
New taxon	Genus	*Atsuirnavirus*		
New taxon	Species	*Atsuirnavirus caloris*	hot spring RNA virus 1	RNA1: BTCN01000001; RNA2: BTCN01000005

*Source/full text: https://ictv.global/ictv/proposals/2024.039B.Artimaviricota_np.zip.

## 2024.040B.Sharonstreetvirus_1ns

**Title:** Create a new species *Sharonstreetvirus xiamensis* (*Caudoviricetes*)

**Author:** Liu H-T (15844493757@163.com)

**Summary**

**Taxonomic rank(s) affected**
Species.
**Description of current taxonomy**
Not currently classified.
**Proposed taxonomic change(s)**
Create a new species *Sharonstreetvirus xiamensis* for phage phiA034 (OP792756) isolated from aquaculture water in Xiamen.
**Justification**
ViPTree analysis classifies phiA034 as a new species the genus *Sharonstreetvirus.*
**Submitted**: 25 April 24

**Table 67.** *Sharonstreetvirus*, 1 new taxon*

**Table:** 

Operation	Rank	New taxon name	Virus name	Exemplar
New taxon	Species	*Sharonstreetvirus xiamensis*	Aeromonas phage phiA034	OP792756

*Source/full text: https://ictv.global/ictv/proposals/2024.040B.Sharonstreetvirus_1ns.zip.

## 2024.041B.Camvirus_2ns

**Title:** Create two new species *– Camvirus vanseggelen* and *Camvirus verabelle* (subfamily *Arquatrovirinae*, class *Caudoviricetes*).

**Authors:** Kempff A (annabelkempff@gmail.com), van Neer V (vanneervera@gmail.com), Ongenae V (v.m.a.ongenae@biology.leidenuniv.nl), Rozen DE, Briegel A, Claessen D

**Summary**

**Taxonomic rank(s) affected**
Realm *Duplodnaviria,* kingdom *Heunggongvirae,* phylum *Uroviricota,* class *Caudoviricetes.*
**Description of current taxonomy**
The genus *Camvirus* is classified within the subfamily *Arquatrovirinae,* class *Caudoviricetes.*
**Proposed taxonomic change(s)**
Create two new species in the genus *Camvirus.*
**Justification**
We have defined two new species, *Camvirus vanseggelen* and *Camvirus verabelle,* based upon phages isolated for host *Streptomyces coelicolor* from soil samples in the Netherlands at longitude N52°23′31″ and latitude E4°34′49″. *Camvirus vanseggelen* contains a linear dsDNA genome of 50 426 bp (65.5% G+C) encoding 73 proteins. *Camvirus verabelle* contains a linear dsDNA genome of 49 832 bp (65.0% G+C) encoding 73 proteins. These new species can be included in the genus *Camvirus* together with Streptomyces phage Alsaber, Streptomyces phage Amela, Streptomyces phage phiCAM, Streptomyces phage Endor1, Streptomyces phage Endor2, Streptomyces phage Joe, Streptomyces phage Saftant and Streptomyces phage Sitrop.
**Submitted:** 02 April 24

**Table 68.** *Camvirus*, 2 new taxa*

**Table:** 

Operation	Rank	New taxon name	Virus name	Exemplar
New taxon	Species	*Camvirus vanseggelen*	Streptomyces phage Vanseggelen	OQ970438
New taxon	Species	*Camvirus verabelle*	Streptomyces phage Verabelle	OQ970439

*Source/full text: https://ictv.global/ictv/proposals/2024.041B.Camvirus_2ns.zip.

## 2024.042B.Lacfervirus_1ng_1ns

**Title:** Create one new genus, *Lacfervirus,* in the class *Caudoviricetes*

**Authors:** Qiannan Wen, Xia Chen (chenxia8280@163.com)

**Summary**

**Taxonomic rank(s) affected**
Realm *Duplodnaviria,* kingdom *Heunggongvirae,* phylum *Uroviricota,* class *Caudoviricetes.*
**Description of current taxonomy**
The virus classified in this proposal does not have a current taxonomic assignment.
**Proposed taxonomic change(s)**
We propose a new genus, *Lacfervirus,* including a single species for the virus Lactobacillus phage LFP01 in the class *Caudoviricetes*.
**Justification**
The genome of Lactobacillus phage LFP01 has very low homology to all other bacteriophage sequences in the GenBank database. A complete genome sequence comparison using the BLASTn method revealed that the genome of Lactobacillus phage LFP01 had a maximum nucleotide identity of 92.83 and 55% coverage with Lactobacillus phage LF1. The low homology indicated that the newly sequenced bacteriophage likely represents a new genus with a single species.
**Submitted:** 05 June 23

**Table 69.** *Lacfervirus*, 2 new taxa*

**Table:** 

Operation	Rank	New taxon name	Virus name	Exemplar
New taxon	Genus	*Lacfervirus*		
New taxon	Species	*Lacfervirus LFP01*	Lactobacillus virus LFP01	OR048821

*Source/full text: https://ictv.global/ictv/proposals/2024.042B.Lacfervirus_1ng_1ns.zip.

## 2024.043B.Cystoviridae_6ng_2nsp_1rng_7rnsp

**Title:** Rename and split an existing genus of the family *Cystoviridae* (*Vidaverviricetes: Mindivirales*)*,* rename seven virus species, create two new species and genera

**Authors:** Poranen MM (minna.poranen@helsinki.fi), Mäntynen S

**Summary**

**Taxonomic rank(s) affected**
The proposal affects species and genus ranks under the family *Cystoviridae*.
**Description of current taxonomy**
The family *Cystoviridae* currently includes one genus *Cystovirus* and seven species, *Cystovirus phi6, Cystovirus phi8, Cystovirus phi12, Cystovirus phi13, Cystovirus phi2954, Cystovirus phiNN* and *Cystovirus phiYY. Cystoviridae* is the only family of the order *Mindivirales* and the class *Vidaverviricetes* that belongs to the phylum *Duplornaviricota* (*Orthornavirae, Riboviria*). The other classes in this phylum are *Resentoviricetes* and *Chrymotiviricetes*.
**Proposed taxonomic change(s)**
We propose a new name for the genus *Cystovirus* and its splitting into five genera. Due to the introduction of the new genera, we propose the renaming of all the current species. In addition, we propose to create two new species and two additional new genera in the family *Cystoviridae*.
**Justification**
Seven new dsRNA bacteriophage isolates have been identified and are now proposed to be taxonomically classified to create two new species. Sequence comparisons of these viruses and previously classified dsRNA bacteriophages of the genus *Cystovirus* justify the splitting of the genus *Cystovirus* and the creation of seven genera in the family *Cystoviridae*. The genus *Cystovirus* is renamed to distinguish the name stems of genus and family rank.
**Submitted**: 21 June 24; **Revised**: 30 September 24

**Table 70.** *Cystoviridae*, 5 move; rename taxa*

**Table:** 

Operation	Rank	New taxon name	Old parent taxon	New parent taxon	Old taxon name
Move; rename taxon	Species	*Alphacystovirus phi8*	*Cystovirus*	*Alphacystovirus*	*Cystovirus phi8*
Move; rename taxon	Species	*Betacystovirus phi12*	*Cystovirus*	*Betacystovirus*	*Cystovirus phi12*
Move; rename taxon	Species	*Gammacystovirus phi13*	*Cystovirus*	*Gammacystovirus*	*Cystovirus phi13*
Move; rename taxon	Species	*Gammacystovirus phiYY*	*Cystovirus*	*Gammacystovirus*	*Cystovirus phiYY*
Move; rename taxon	Species	*Deltacystovirus phi2954*	*Cystovirus*	*Deltacystovirus*	*Cystovirus phi2954*

**Table 71.** *Cystoviridae*, 4 new taxa*

**Table:** 

Operation	Rank	New taxon name	Virus name	Exemplar
New taxon	Genus	*Epsiloncystovirus*		
New taxon	Species	*Epsiloncystovirus phiNY*	Microvirgula phage phiNY	MW471133; MW471134; MW471135
New taxon	Genus	*Zetacystovirus*		
New taxon	Species	*Zetacystovirus CAP*	Acinetobacter phage CAP7	MZ558516; MZ558517; MZ558518

**Table 72.** *Cystoviridae*, 3 rename taxa*

**Table:** 

Operation	Rank	New taxon name	Previous taxon name
Rename taxon	Genus	*Orthocystovirus*	*Cystovirus*
Rename taxon	Species	*Orthocystovirus phi6*	*Cystovirus phi6*
Rename taxon	Species	*Orthocystovirus phiNN*	*Cystovirus phiNN*

**Table 73.** *Cystoviridae*, 1 split taxa, 4 new taxa*

**Table:** 

Operation	Rank	Old taxon	New taxon 1	New taxon 2	New taxon 3	New taxon 4
Split taxon	Genus	*Cystovirus*	*Alphacystovirus*	*Betacystovirus*	*Gammacystovirus*	*Deltacystovirus*

*Source/full text: https://ictv.global/ictv/proposals/2024.043B.Cystoviridae_6ng_2nsp_1rng_7rnsp.zip.

## 2024.044B.Felixviridae_1nf_1nsf_2ng_1mg_2ns

**Title:** Create one new family (*Felixviridae*)*,* including one new subfamily (*Maevirinae*)*,* three genera (two new: *Nakavirus, Chronisvirus;* one existent*: Certevirus*)*,* including two new species (*Nakavirus sapi* and *Chronisvirus chronis*).

**Authors:** Nobrega F.L. (f.nobrega@soton.ac.uk), Rothschild-Rodriguez, D., Lambon, K.

**Summary**

**Taxonomic rank(s) affected**
*Duplodnaviria; Heunggongvirae; Uroviricota; Caudoviricetes.*
**Description of current taxonomy**
Unclassified *Caudoviricetes*.
**Proposed taxonomic change(s)**
We propose a new family, *Felixviridae,* which includes the new subfamily, *Maevirinae,* and three genera: *Nakavirus* (new), *Chronisvirus* (new) and *Certevirus* (already existent). The *Nakavirus* and *Chronisvirus* genera include at least one newly proposed species each, *Nakavirus sapi* (for Klebsiella phage Roth C and Klebsiella phage Roth D), and *Chronisvirus chronis* (for Klebsiella phage vB_Kpn_Chronis), respectively.
**Justification**
Phages assigned to this new family have not been taxonomically characterised, with only one previously cultured *Klebsiella* phage relative, vB_Kpn_Chronis, and a Protoea phage, PdC23. We isolated 53 phages as part of the Klebsiella Phage Collection, two of which, RothC and RothD, could not be assigned to any existing viral families, leading us to propose a new family, *Felixviridae*. Additionally, we found representatives of this family to be present in metagenomes originating from human stool samples, a proxy for the human gut microbiota, and in metagenomic sequences within the Gut Phage Database. Genomic analyses suggest that the family *Felixviridae* includes at least one subfamily, *Maevirinae,* and three genera, *Certevirus* and two new genera *Nakavirus* and *Chronisvirus*. The *Nakavirus* genus includes species for Klebsiella phage Roth C and Klebsiella phage Roth D. High similarity between these viruses places them both in the proposed species *Nakavirus sapi*. The *Chronisvirus* genus includes the species *Chronisvirus chronis* for phage vB_Kpn_Chronis.
**Submitted**: 19 June 24; **Revised**: 27 June 24

**Table 74.** *Felixviridae*, 6 new taxa*

**Table:** 

Operation	Rank	New taxon name	Virus name	Exemplar
New taxon	Family	*Felixviridae*		
New taxon	Subfamily	*Maevirinae*		
New taxon	Genus	*Chronisvirus*		
New taxon	Species	*Chronisvirus chronis*	vB_Kpn_Chronis	MN013086
New taxon	Genus	*Nakavirus*		
New taxon	Species	*Nakavirus sapi*	Klebsiella phage RothC	PP934563

**Table 75.** *Felixviridae*, 1 move taxon*

**Table:** 

Operation	Rank	Taxon name	New parent taxon	Old parent taxon
Move taxon	Genus	*Certevirus*	*Felixviridae*	*Caudoviricetes*

*Source/full text: https://ictv.global/ictv/proposals/2024.044B.Felixviridae_1nf_1nsf_2ng_1mg_2ns.zip.

## 2024.045B.Autographivirales

**Title:** Promoting the family *Autographiviridae* to create one new order, *Autographivirales,* with four new families, four new subfamilies, 93 new genera and 607 new species (*Duplodnaviria, Caudoviricetes*).

**Authors:** Turner D (dann2.Turner@uwe.ac.uk), Carrillo D, Lood C, Ely B, Lehman SM, Dutilh B, Kropinski AM, Lavigne R, Adriaenssens EM, Millard AD

**Summary**

**Taxonomic rank(s) affected**
Realm: *Duplodnaviria*; kingdom: *Heunggongvirae*; phylum: *Uroviricota*; class: *Caudoviricetes.*
**Description of current taxonomy**
The family *Autographiviridae* was established under taxonomic proposal 2019.103B.
**Proposed taxonomic change(s)**
We propose
The establishment of a new order, *Autographivirales,* containing four new families.
The creation of four new subfamilies.
The creation of 93 new genera.
The creation of 610 new species.
Abolition of 21 species.
**Justification**
The proposed order forms a single deep-branching clade in tBLASTx distance analysis, reflected in core gene maximum-likelihood phylogeny. The proposed families form monophyletic clusters in proteome-based analyses and each share a number of core orthologous genes.
**Submitted:** 21 June 24; **Revised:** 30 September 24

**Table 76.** *Autographivirales*, 708 new taxa*. Table too large, see supplementary information sheet supp_info_tab_76

**Table 77.** *Autographivirales*, 60 move taxa*. Table too large, see supplementary information sheet supp_info_tab_77

**Table 78.** *Autographivirales*, 21 abolish taxa*

**Table:** 

Operation	Rank	Abolished taxon name
Abolish taxon	Species	*Friunavirus SWHAb1*
Abolish taxon	Species	*Friunavirus SWHAb3*
Abolish taxon	Species	*Phikmvvirus NFS*
Abolish taxon	Species	*Phikmvvirus PT2*
Abolish taxon	Species	*Vectrevirus cee*
Abolish taxon	Species	*Maculvirus OWB*
Abolish taxon	Species	*Murciavirus CB5A*
Abolish taxon	Species	*Kaohsiungvirus AS51*
Abolish taxon	Species	*Cuernavacavirus RHEph09*
Abolish taxon	Species	*Atuphduovirus atuph03*
Abolish taxon	Species	*Przondovirus KpV766*
Abolish taxon	Species	*Teseptimavirus YpsPG*
Abolish taxon	Species	*Teseptimavirus YpPY*
Abolish taxon	Species	*Helsettvirus fPS53*
Abolish taxon	Species	*Teetrevirus T7M*
Abolish taxon	Species	*Berlinvirus Yepe2*
Abolish taxon	Species	*Berlinvirus Yepf*
Abolish taxon	Species	*Kayfunavirus EcpYZU01*
Abolish taxon	Species	*Pektosvirus PP81*
Abolish taxon	Species	*Ghunavirus Psa17*
Abolish taxon	Species	*Friunavirus AB3*

**Table 79.** *Autographivirales*, 14 move; rename taxa*

**Table:** 

Operation	Rank	New taxon name	New parent taxon	Old taxon name
Move; rename taxon	Species	*Rodentiumvirus LL11*	*Rodentiumvirus*	*Vectrevirus LL11*
Move; rename taxon	Species	*Rodentiumvirus CrRp3*	*Rodentiumvirus*	*Vectrevirus CrRp3*
Move; rename taxon	Species	*Nerthusvirus achelous*	*Nerthusvirus*	*Uliginvirus achelous*
Move; rename taxon	Species	*Nerthusvirus nerthus*	*Nerthusvirus*	*Uliginvirus nerthus*
Move; rename taxon	Species	*Nerthusvirus alpheus*	*Nerthusvirus*	*Uliginvirus alpheus*
Move; rename taxon	Species	*Njordvirus njord*	*Njordvirus*	*Uliginvirus njord*
Move; rename taxon	Species	*Ebriosvirus ebrios*	*Ebriosvirus*	*Teseptimavirus ebrios*
Move; rename taxon	Species	*Ebriosvirus IME15*	*Ebriosvirus*	*Teseptimavirus IME15*
Move; rename taxon	Species	*Hennigervirus shl2*	*Hennigervirus*	*Ghunavirus shl2*
Move; rename taxon	Species	*Hennigervirus PPPL1*	*Hennigervirus*	*Ghunavirus PPPL1*
Move; rename taxon	Species	*Hennigervirus henninger*	*Hennigervirus*	*Ghunavirus henninger*
Move; rename taxon	Species	*Unosvirus UNOSLW1*	*Unosvirus*	*Pifdecavirus UNOSLW1*
Move; rename taxon	Species	*Pfluvirus PFP1*	*Pfluvirus*	*Pifdecavirus PFP1*
Move; rename taxon	Species	*Pfluvirus pv22PfluR64PP*	*Pfluvirus*	*Pifdecavirus pv22PfluR64PP*

**Table 80.** *Autographivirales*, 2 rename taxa*

**Table:** 

Operation	Rank	New taxon name	Previous taxon name
Rename taxon	Species	*Ghunavirus gv17A*	*Ghunavirus 17A*
Rename taxon	Species	*Warsawvirus wv3MF5*	*Warsawvirus 3MF5*

**Table 81.** *Autographivirales*, 1 promote taxon*

**Table:** 

Operation	New taxon name	Old rank	New rank
Promote taxon	*Autographivirales*	Family	Order

*Source/full text: https://ictv.global/ictv/proposals/2024.045B.Autographivirales.zip.

## Supplementary material

10.1099/jgv.0.002111 Uncited Table S1. *Andersonviridae*, 100 new taxa*

10.1099/jgv.0.002111 Uncited Table S2. *Ferrettivirinae*, 42 new taxa*. supp_info_tab_25*Source / full text: <https://ictv.global/ictv/proposals/2024.013B.Ferrettivirinae_1nsf_3ng_38ns.zip>;

10.1099/jgv.0.002111 Uncited Table S3. *Caudoviricetes*, 96 rename taxa*

10.1099/jgv.0.002111 Uncited Table S4. *Autographivirales*, 708 new taxa*

10.1099/jgv.0.002111 Uncited Table S5. *Autographivirales*, 60 move taxa*

## Abbreviations

ICTV: International Committee on Taxonomy of Viruses
RdRP: RNA-directed RNA polymerase
